# Uncovering the drivers of anuran age structure

**DOI:** 10.1186/s12983-026-00630-5

**Published:** 2026-08-27

**Authors:** Xiuping Wang, Wenyi Zhang, Bin Wang, Jianping Jiang, Meihua Zhang, Bingjun Dong

**Affiliations:** 1 Chengdu Institute of Biology, Chinese Academy of Sciences, Chengdu, 610213 China; 2 College of Life Sciences, Shenyang Normal University, Shenyang, 110034 China

**Keywords:** Lifespan, Average age, Age at sexual maturity, Climate change, Environmental stress, Age prediction

## Abstract

Age structure provides a vital perspective for understanding population ecology, evolutionary biology, and conservation biology. Here, we compiled data on maximum age, average age, and age at sexual maturity for 184 anuran species across 22 families from published literature. We evaluated the effects of climate factors, geographical gradients, habitat types, and body size on age-structure traits. Furthermore, we projected future trends in age structure under various climate change scenarios. Our results indicate that broad habitat categories did not significantly influence age-related traits, likely due to the buffering capacity of microhabitats, which mitigates climate-related differences across habitats. In contrast, age traits exhibited sex-specific responses to environmental drivers, potentially reflecting divergent reproductive energy allocation strategies and metabolic trade-offs between males and females. Future projections suggest that climate change will lead to a widespread decline in both lifespan and average age across anurans, resulting in a shift toward younger populations with shorter lifespans and a general trend toward smaller body size. These findings deepen our understanding of how anurans respond to environmental changes and provide a scientific foundation for developing effective conservation strategies.

**Supplementary Information:**

The online version contains supplementary material available at https://doi.org/10.1186/s12983-026-00630-5.

## Background

Age structure, defined as the distribution of individuals across different age groups, plays a crucial role in biodiversity conservation, particularly in the context of accelerating global climate change [1, 2]. As a key life-history trait and demographic indicator, age structure offers valuable insights into population health, dynamics, and long-term viability [3, 4]. Furthermore, it reveals adaptive variation in developmental patterns and reproductive strategies, and highlights interspecific differences in sensitivity to environmental stressors [5, 6]. Age structure is shaped by a range of factors, and analyzing age-related parameters can help uncover the underlying mechanisms of population or species declines. Such research is critical for assessing the impacts of climate change, evaluating extinction risk, and developing targeted conservation measures [7–9].

Climate change, encompassing global warming, more frequent extreme weather events, altered precipitation regimes, and rising sea levels, poses severe threats to global biodiversity. Climate projections suggest that over the next 50 years, the frequency and intensity of heatwaves will increase substantially, driving environmental temperatures toward or beyond the thermal tolerance limits of ectothermic animals [10]. Rising temperatures can induce oxidative stress and cellular damage, accelerating physiological aging processes. Concurrent changes in temperature and precipitation may further disrupt developmental processes and reduce adult survival rates [11]. Additionally, rising global temperatures are altering hydrological cycles, leading to more frequent heavy rainfall events and greater variability in precipitation patterns, including both droughts and floods. These disruptions undermine the stability of breeding habitats, degrade water quality [12], and expand the transmission windows for infectious pathogens [13]. Such drivers often act synergistically, imposing multifaceted stresses that detrimentally affect species physiology [14], phenology [15], distribution [16], and ultimately elevate extinction risk [17]. However, owing to pronounced spatiotemporal variation in the magnitude of individual climatic factors, the interactions among these stressors are highly complex and remain insufficiently understood [18, 19]. Alongside habitat loss, pollution, and diseases, climate change is widely recognized as one of the most critical threats to tetrapods, particularly ectothermic species [20]. Amphibians are especially vulnerable due to their high environmental sensitivity, permeable skin, small body size, and limited dispersal ability [21]. According to the IUCN Red List of Threatened Species, at least 41% of known amphibian species are currently threatened with extinction, the highest proportion recorded among vertebrate groups [22]. Assessing climate-driven amphibian declines therefore requires an integrated, multi-scale analysis of how climatic factors shape key life-history traits [23].

Amphibians exhibit high sensitivity to environmental conditions, with their population age structure particularly shaped by geographical gradients [24, 25]. High-elevation environments expose organisms to multiple interacting selective pressures, including hypoxia, low temperatures, and intense ultraviolet radiation, which directly influence individual survival and reproductive performance [26]. Such conditions are often associated with reduced intrinsic mortality, delayed sexual maturity, and extended lifespan [27–29]. Across many anuran species, both age structure and body size vary predictably along elevational gradients, with high-elevation populations frequently exhibiting larger body sizes and older age distributions [30]. Cold environments at high elevations slow life-history tempos, while prolonged hibernation reduces metabolic rates and external predation pressure, thereby limiting oxidative damage and mutation accumulation [31, 32]. Collectively, these processes contribute to lifespan extension. This pattern has been observed in both amphibians and reptiles [33, 34]. Amphibians inhabiting high-elevation or high-latitude regions generally experience shortened activity seasons, slower growth rates, and reduced metabolic demands. However, climate warming is altering high-elevation hydrological regimes, leading to reduced snowfall and increased ultraviolet radiation. These changes constrain the availability of suitable amphibian habitats, diminish reproductive success, and ultimately exert profound effects on breeding period length and lifespan [35]. Furthermore, under significantly warmer conditions, high-latitude species tend to initiate reproduction earlier [36]. Nevertheless, how amphibian population age structure will respond to future climate scenarios—and the mechanisms underlying these responses—remains poorly understood.

Habitat loss is one of the most critical threats to amphibian populations worldwide [37]. Owing to their complex life histories, amphibians are highly dependent on habitat conditions, which directly regulate physiological conditions, growth trajectories, and survival rates [38]. Amphibians exhibit considerable developmental plasticity in growth in response to environmental pressures such as predation pressure, intraspecific competition, and the desiccation of aquatic habitats. Species inhabiting stable aquatic environments generally experience lower mortality risk and exhibit higher growth potential. Consequently, anurans occupying permanent or semi-permanent water bodies often prolong their developmental period to attain larger body sizes and enhance survival probability [39]. However, the increasing desiccation of aquatic habitats driven by global warming is profoundly disrupting these adaptive strategies [40]. Vegetation, which responds strongly to climate change, plays a key role in habitat integrity [41]. Severe drought events can cause widespread tree mortality and canopy loss, directly degrading the habitats for arboreal amphibian species [42]. Similarly, fossorial amphibians exhibit high vulnerability to climate-driven environmental change [43]. Altered precipitation patterns and rapidly declining soil moisture under global warming pose severe threats to these subterranean species [44]. Although understanding habitat availability is fundamental for developing effective conservation strategies, such knowledge remains limited for the majority of amphibian species.

Body size is a pivotal trait shaping biodiversity patterns from regional to global scales, profoundly influencing ecological, evolutionary and demographic processes [45]. It regulates multiple aspects of species biology, including reproductive output, physiological performance, and population dynamics [46]. As a central indicator of resource acquisition and allocation, body size is closely associated with lifespan [47]. This relationship may be attributed to differences in metabolic efficiency and anti-predator adaptations [48], with larger body size generally conferring reduced predation risk [49]. Under global warming, smaller body size may confer selective advantages in a warmer environment due to a higher surface area-to-volume ratio that facilitates heat dissipation [50]. Consequently, anurans may exhibit a trend toward reduced body size, potentially lowering temperature-related extrinsic mortality and thereby reshaping population-level age structure [51]. However, the increasing frequency of heatwaves and extreme climatic events may simultaneously accelerate physiological aging processes. Given the pervasive influence of body size on organismal fitness [52], understanding the ecological correlates of this multidimensional trait remains a central objective of life-history theory [53].

In this study, we examine how multiple factors—including climate, geographical gradients, habitat conditions, and body size—interact to shape the age structure of anurans. We quantify the relative contributions of climate variables that have direct and significant effects on age structure, and integrate existing anuran age data with future climate projections to assess potential shifts in age structure due to ongoing environmental changes. This research aims to establish a mechanistic link between environmental stressors and age structure, and identifies key parameters that drive the ecological processes underlying the global decline of amphibians. By doing so, this study aims to enhance our understanding of anuran biological traits and life-history strategies, and to provide insights into potential changes in age structure in the context of global climate change, with important implications for conservation biology.

## Methods

### Data collection

We compiled life-history traits and geographic data for 184 anuran species from wild populations using literature sources, representing 22 families worldwide [54]. The dataset includes lifespan, age at sexual maturity, average age, mean snout–vent length (mean SVL), maximum snout–vent length (max SVL), geographic coordinates (longitude and latitude), elevation, and habitat types. We only included age estimates that were obtained via skeletochronology [55]. Lifespan was defined as the maximum recorded age documented in the literature for each record; age at sexual maturity refers to the minimum age at first reproduction; and average age represents the mean age of all individuals within the sampled population at the time of sampling. For species with multiple records (e.g., from different populations or studies), species-level averages for age and SVL were calculated [56]. Elevation was represented by the midpoint value between the highest and lowest recorded elevations for each species [57]. Habitat types were categorized as semi-aquatic, terrestrial, fossorial, or arboreal according to the classification systems of Fei et al. [58] and AmphibiaWeb (https://amphibiaweb.org).

To assess the effects of climatic factors on anuran age structure variation, we obtained 19 bioclimatic variables (BIO1-BIO19) from WorldClim [59] (http://www.worldclim.org/). This dataset encompasses both historical climate records and future projections derived from the CMIP6 ensemble under three Shared Socioeconomic Pathways (SSPs): SSP126 (low radiative forcing), SSP245 (intermediate), and SSP585 (high radiative forcing). Future climate projections were retrieved for two time horizons: 2041–2060 (mid-century) and 2081–2100 (end-century). In addition, four UV-B variables were acquired from the glUV database [60] (https://www.ufz.de/gluv/). Species occurrence coordinates were processed in ArcMap v 10.8 (Esri, Redlands, CA, USA), and environmental data were extracted at a spatial resolution of 30 arc-seconds. To mitigate multicollinearity among the 23 initial climatic predictors, pairwise correlations were evaluated, and variables with |Pearson *r*| ≥ 0.8 were excluded [61]. After this screening process, four key variables were retained for subsequent analysis: annual mean temperature (BIO1), temperature seasonality (BIO4), annual precipitation (BIO12), and annual mean UV-B (UVB1).

### Phylogenetic relationship reconstruction

**Fig. 1:**
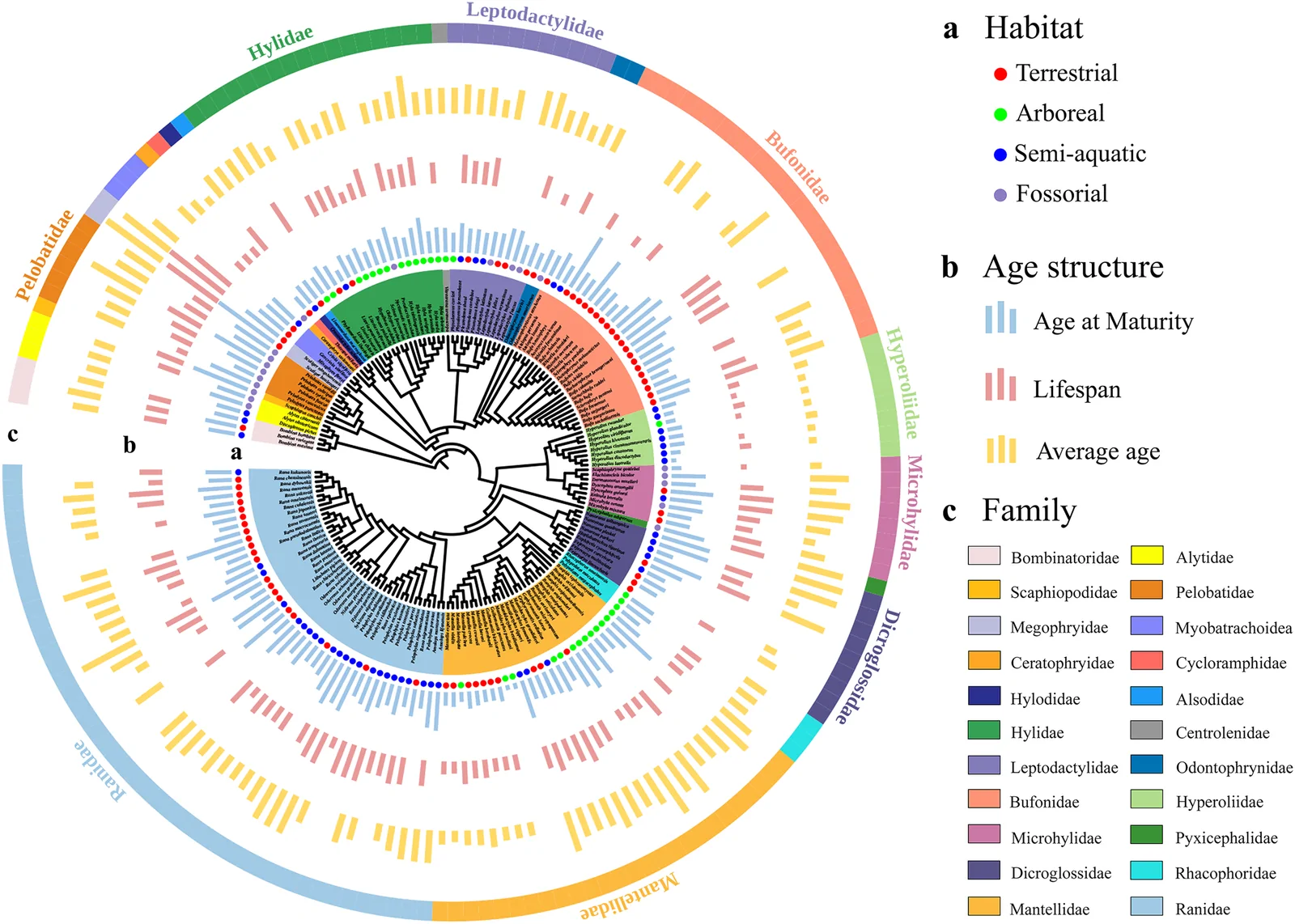
Phylogenetic relationships among the 184 anuran species included in this study. (**a**) Colored circles represent habitat types; (**b**) bar charts adjacent to tips represent age-structure variation, and (**c**) colored rectangles denote taxonomic families. The order of families in the legend corresponds to their first occurrence clockwise from the opening of the phylogeny

To reconstruct the phylogenetic relationships, we pruned and reconciled the amphibian phylogenetic tree published by Jetz and Pyron to match the species in our dataset [62]. Of the 184 species, 7 species were represented by congeneric or closely related taxa as phylogenetic proxies due to their absence in the original tree. Subsequently, we generated a maximum clade credibility (MCC) tree with mean node heights using TreeAnnotator v1.10.4 [63], and the resulting tree was used for all subsequent phylogenetic analyses (Fig. 1).

### Phylogenetic signal detection

We estimated the phylogenetic signal for lifespan (*n* = 174), age at sexual maturity (*n* = 126), and average age (*n* = 147) using Pagel’s λ to evaluate the influence of evolutionary history on age-related variation. Values approaching 1 indicate strong phylogenetic dependence (trait evolution consistent with Brownian motion), whereas values approaching 0 suggest phylogenetic independence [64].

### Phylogenetic generalized least squares modeling

To account for phylogenetic non-independence among species and assess the effects of predictor variables on anuran age structure, we conducted phylogenetic generalized least squares (PGLS) analyses using the “caper” package v1.0.3 [65]. Sample sizes varied by trait and sex, lifespan (174; males: 173, females: 78), average age (147; males: 147, females: 142), and age at sexual maturity (126; males: 126, females: 118). PGLS incorporates phylogenetic relationships into the error structure via a variance-covariance matrix to correct for residual correlations. All continuous predictors were standardized (z-score) prior to modeling to facilitate comparison of effect sizes. We first assessed collinearity among predictors using variance inflation factors (VIF), variables with VIF > 5 were considered to exhibit strong collinearity [66]. Results indicated strong collinearity among climate variables, elevation, and latitude. To mitigate this issue and identify robust drivers of age variation, we constructed two distinct model sets following Amat and Meiri [67]. Model 1 included elevation, latitude, max SVL, and habitat types as predictors. Model 2 incorporated four climatic variables (i.e., annual mean temperature, temperature seasonality, annual precipitation, annual mean UV-B), along with max SVL and habitat types. Mean SVL was used as the body-size predictor for the average-age models. For each model set, we began with a global model containing all main effects and two-way interactions. We then performed backward stepwise model selection based on *p*-values (α = 0.05) to derive the best-supported reduced model.

### Phylogenetic path analysis

To investigate the potential drivers of age structure variation, we conducted phylogenetic path analysis (PPA) on 65 species. All continuous variables were standardized (z-score) prior to analysis. Based on theoretical expectations and preliminary PGLS results, we constructed two sets of candidate models. The baseline ecological model set comprised fifteen models incorporating lifespan, age at sexual maturity, average age, max SVL, elevation, and latitude. The climate-extended model set comprised fifteen models that additionally integrated annual mean temperature, temperature seasonality, annual precipitation, and annual mean UV-B. All models were developed following the analytical framework of Hardenberg and Gonzalez-Voyer [68]. Model fit was assessed using the small-sample-size corrected C-statistic information criterion (CICc). We ranked models by CICc and averaged those with ΔCICc ≤ 2 to account for model selection uncertainty and obtain robust parameter estimates. Model construction and validation adhered to the standardized protocol outlined by van der Bijl [69]. Model fit indices (e.g., CICc, R²) and statistical significance were reported in accordance with established guidelines.

### Contribution of climatic factors and future age predictions

To quantify the independent contributions of significant climatic variables to age-related traits and body size, we conducted hierarchical partitioning analysis using the “hier.part” package v1.0-5 [70]. Climate variables were standardized via z-score normalization to account for scale differences [71]. Based on the significant predictors identified above, we built four separate random forest models to predict lifespan, average age, age at sexual maturity, and max SVL using the “ranger” algorithm v0.17.0 [72]. Model performance was evaluated via out-of-bag (OOB) error and cross-validation, with root mean square error (RMSE) and coefficient of determination (R²) as key metrics. Predictions under future climate scenarios were compared against historical baselines following the approach of Cutler et al. [73]. All trait responses were expressed as relative changes (%) with respect to historical means. These analyses included 106 species. All analyses were performed in R version 4.4.2 [74].

## Results

### Phylogenetic signal detection

All examined traits (i.e., lifespan (*n* = 174), age at sexual maturity (*n* = 126), and average age (*n* = 147)) across overall, male, and female datasets showed significant phylogenetic signal (*p* < 0.05; Table 1), indicating that evolutionary history constrains these life-history characteristics.

**Table 1:** Phylogenetic signal estimates for age-structure traits

	K	λ	*p*
**Lifespan**			
Total	0.223	0.759	< 0.001
Male	0.244	0.769	< 0.001
Female	0.157	0.592	< 0.001
**Age at sexual maturity**			
Total	0.202	0.745	< 0.01
Male	0.212	0.603	< 0.01
Female	0.175	0.727	< 0.01
**Average age**			
Total	0.188	0.766	< 0.05
Male	0.139	0.719	< 0.05
Female	0.250	0.764	< 0.05

Notes: *p* values indicate the statistical significance of Pagel’s λ

### Phylogenetic generalized least squares

After accounting for phylogenetic non-independence, the best-fit models retained multiple significant predictors and key interaction terms (for complete model please see Supplementary Tables S1-S2, Additional File 1). Geographic model results (Table 2) showed that male lifespan was positively influenced by latitude and max SVL. Combined-sex and female lifespan were positively affected by latitude and the interaction between elevation and max SVL, but negatively affected by the interaction between latitude and elevation. Age at sexual maturity was positively associated with the interaction between latitude and max SVL across all groups. Similarly, average age showed a positive response to the interaction between latitude and mean SVL.

**Table 2:** Minimal adequate phylogenetic generalized least squares (PGLS) model for geographic variables (elevation and latitude) and body size

Data_Set	Parameter	Estimate ± SE	T	*p*	95% CI
**Lifespan**					
Total	Intercept	-0.665 ± 0.188	-3.539	0.001	(NA, 0.699)
	Latitude	0.923 ± 0.124	7.452	< 0.001	
	Latitude: Elevation	-0.213 ± 0.045	-4.769	< 0.001	
	Elevation: max SVL	0.194 ± 0.038	5.100	< 0.001	
Males	Intercept	-0.421 ± 0.144	-2.931	< 0.001	(NA, 0.517)
	Latitude	0.246 ± 0.055	4.501	< 0.001	
	max SVL	0.462 ± 0.096	4.803	< 0.001	
Females	Intercept	-0.722 ± 0.220	-3.283	0.002	(NA, 0.618)
	Latitude	0.960 ± 0.144	6.675	< 0.001	
	Latitude: Elevation	-0.236 ± 0.050	-4.726	< 0.001	
	Elevation: max SVL	0.213 ± 0.042	5.057	< 0.001	
**Age at sexual maturity**					
Total	Intercept	-0.234 ± 0.169	-1.387	0.169	(0275, 0.959)
	Latitude: max SVL	0.208 ± 0.055	3.759	< 0.001	
Males	Intercept	-0.049 ± 0.136	-0.359	0.720	(0.022, 0.868)
	Latitude: max SVL	0.137 ± 0.048	2.878	0.005	
Females	Intercept	-0.104 ± 0.145	-0.719	0.474	(NA, 0.911)
	Latitude: max SVL	0.172 ± 0.048	3.557	< 0.001	
**Average age**					
Total	Intercept	-0.118 ± 0.129	-0.919	0.361	(NA, 0.305)
	Latitude: mean SVL	0.274 ± 0.043	6.315	< 0.001	
Males	Intercept	-0.099 ± 0.087	-1.145	0.255	(NA, 0.410)
	Latitude: mean SVL	0.256 ± 0.033	7.835	< 0.001	
Females	Intercept	-0.069 ± 0.124	-0.556	0.579	(NA, 0.318)
	Latitude: mean SVL	0.264 ± 0.043	6.177	< 0.001	

Notes: CI, confidence interval. *p* values indicate significance levels of model terms

Climatic model results (Table 3) indicated that UVB1 radiation exerted a negative effect on lifespan, although its interaction with temperature seasonality, as well as the interaction between annual precipitation and max SVL had positive effects. Male lifespan was also positively influenced by the interaction between UVB1 and annual precipitation. For the age at sexual maturity, UVB1 and annual precipitation showed a significant positive interactive effect across all datasets (*p* < 0.01). Temperature seasonality delayed sexual maturity in total and female groups, yet in females, it formed significant antagonistic counteractions with both annual precipitation and UVB1, ultimately accelerating sexual maturation. Annual precipitation significantly and negatively affected average age, but its interaction with mean SVL exhibited a strong positive effect (*p* < 0.001). In total and male datasets, annual temperature positively influenced average age, whereas its interaction with annual precipitation exerted a negative effect. Although UVB1 negatively affected average age, it synergistically enhanced this trait when interacting with temperature seasonality or annual precipitation. In the female dataset, the interaction between annual temperature and mean SVL negatively affected average age.

**Table 3:** Minimal adequate phylogenetic generalized least squares (PGLS) model for climatic variables (Bio1, Annual mean temperature; Bio4, Temperature seasonality; Bio12, Annual precipitation, and UVB1, Annual mean UV-B)

Data_Set	Parameter	Estimate ± SE	T	*p*	95%CI
**Lifespan**					
Total	Intercept	1.180 ± 0.563	2.096	0.039	(NA, 0.691)
	UVB1	-0.475 ± 0.129	-3.689	< 0.001	
	UVB1: Bio4	0.062 ± 0.016	3.789	< 0.001	
	Bio12: max SVL	0.129 ± 0.026	4.994	< 0.001	
Males	Intercept	1.621 ± 0.536	3.029	0.003	(NA, 0.402)
	UVB1	-0.757 ± 0.148	-5.102	< 0.001	
	UVB1: Bio4	0.052 ± 0.179	2.908	0.005	
	UVB1: Bio12	0.048 ± 0.021	2.288	0.02	
	UVB1: max SVL	0.132 ± 0.028	4.780	< 0.001	
Females	Intercept	1.402 ± 0.628	2.235	0.028	(NA, 0.575)
	UVB1	-0.509 ± 0.143	-3.565	< 0.001	
	UVB1: Bio4	0.051 ± 0.021	2.445	0.017	
	Bio12: max SVL	0.133 ± 0.029	4.507	< 0.001	
**Age at sexual maturity**					
Total	Intercept	-1.648 ± 0.462	-3.565	0.001	(0.034, 0.934)
	Bio4	0.505 ± 0.108	4.698	0.000	
	Bio12:UVB1	0.056 ± 0.020	2.820	0.001	
Males	Intercept	-0.170 ± 0.158	-1.081	0.284	(0.014, 0.876)
	Bio12:UVB1	0.101 ± 0.031	3.238	0.002	
Females	Intercept	-6.223 ± 1.671	-3.723	0.000	(0.118, 0.900)
	Bio4	3.883 ± 1.054	3.685	0.000	
	Bio4:Bio12	-0.557 ± 0.185	-3.014	0.003	
	Bio4:UVB1	-0.481 ± 0.136	-3.543	0.001	
	Bio12:UVB1	0.487 ± 0.144	3.386	0.001	
**Average age**					
Total	Intercept	18.246 ± 6.466	2.822	0.006	(NA, 0.615)
	Bio1	3.305 ± 1.265	2.614	0.011	
	Bio12	-6.307 ± 2.255	-2.797	0.006	
	UVB1	-6.501 ± 2.165	-3.003	0.004	
	Bio1:Bio12	-1.137 ± 0.434	-2.619	0.011	
	UVB1:Bio4	0.104 ± 0.029	3.572	0.001	
	Bio12:UVB1	2.137 ± 0.742	2.880	0.005	
	Bio12:mean SVL	0.134 ± 0.037	3.647	0.000	
Males	Intercept	32.659 ± 7.735	4.222	0.000	(NA, 0.615)
	Bio1	3.827 ± 1.364	2.806	0.006	
	Bio12	-11.266 ± 2.689	-4.190	0.000	
	UVB1	-10.762 ± 2.527	-4.259	0.000	
	Bio1:Bio12	-1.279 ± 0.467	-2.738	0.008	
	UVB1:Bio4	0.116 ± 0.021	5.556	0.000	
	Bio12:UVB1	3.570 ± 0.867	4.117	0.000	
	Bio12:mean SVL	0.145 ± 0.031	4.677	0.000	
Females	Intercept	0.518 ± 0.188	2.750	0.007	(NA, 0.175)
	Bio12	-0.229 ± 0.092	-2.489	0.015	
	Bio1:mean SVL	-0.085 ± 0.041	-2.079	0.041	
	Bio12:mean SVL	0.178 ± 0.047	3.809	0.000	

Notes: CI, confidence interval. *p* values indicate the significance of model predictors

### Phylogenetic path analysis

**Fig. 2:**
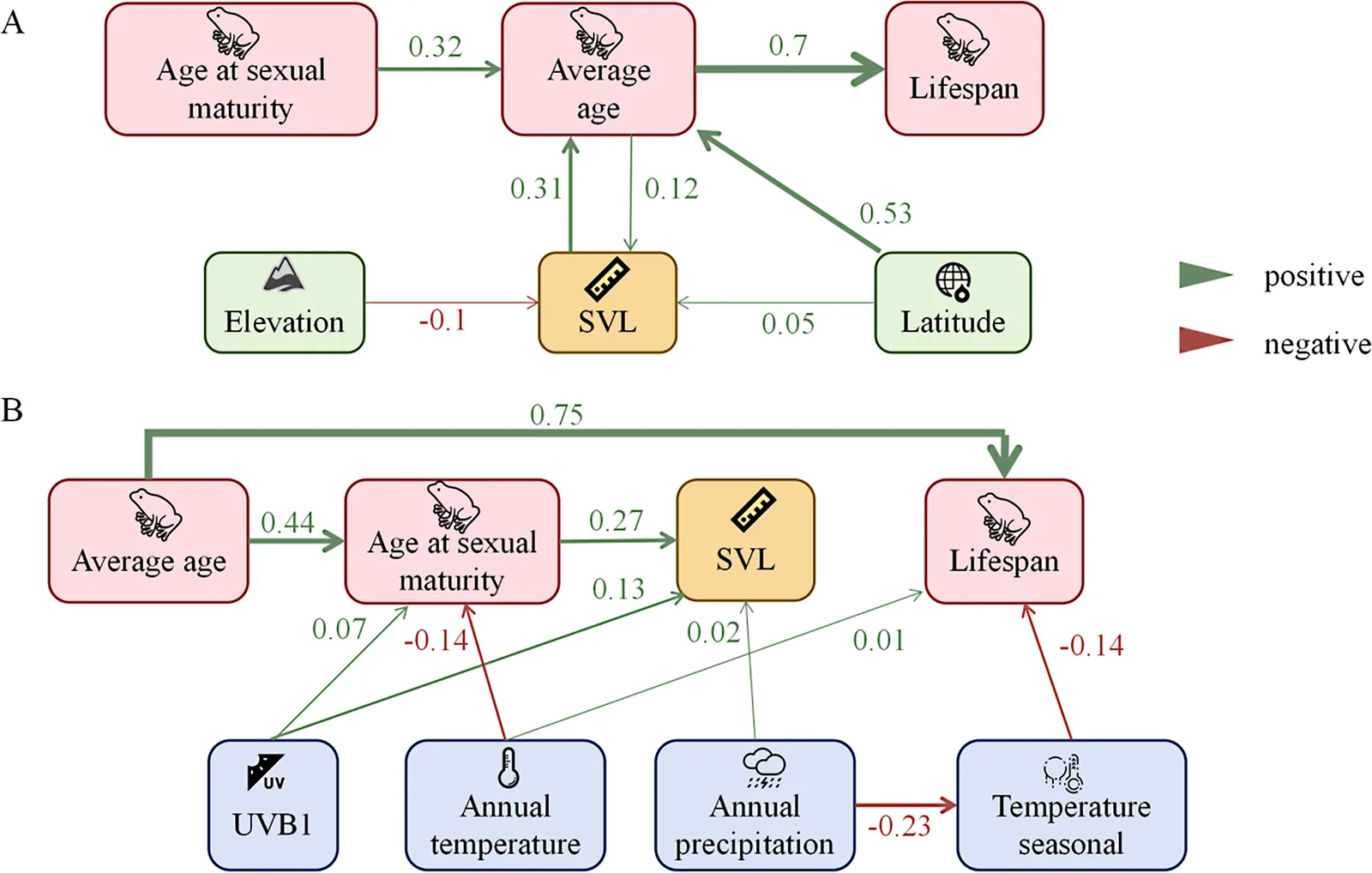
Average best-fitting phylogenetic path models for anurans (ΔCICc ≤ 2). **A** Best-supported model incorporating geographical variables; **B** Best-supported model incorporating climatic variables. Arrows indicate path direction, line width corresponds to the magnitude of standardized regression coefficients, with green and red representing positive and negative relationships, respectively

Phylogenetic path analysis was conducted on 65 species with complete data for all variables. The optimal geographical model supported both direct and indirect effects of max SVL, age structure, elevation and latitude on lifespan (Fig. 2A, Supplementary Table S3, Additional File 1). Age at sexual maturity was positively correlated with average age (0.32), and average age strongly predicted lifespan (0.7), indicating synchronization among life-history traits. Average age and SVL were positively correlated. Elevation negatively correlated with SVL (-0.1), suggesting body size reduction at higher elevations, while the latitude positively correlated with both average age (0.53) and SVL (0.05).

The best climatic model revealed complex interactions among environmental variables influencing age structure (Fig. 2B, Table S4). Lifespan had the strongest positive correlation (0.75) with average age, which was moderately correlated with age at sexual maturity (0.44). The latter also correlated positively with max SVL (0.27). Environmental analyses revealed that UVB1 was weakly positively correlated with age at sexual maturity (0.07) and max SVL (0.13). Annual temperature was negatively correlated with age at sexual maturity (-0.14) and positively with lifespan (0.01). Annual precipitation reduced temperature seasonality (-0.23), and temperature seasonality had a negative effect on lifespan (-0.14).

Overall, phylogenetic path analysis results were consistent with those from PGLS regression.

### Contribution of climatic factors (Fig. 3A)

Hierarchical partitioning revealed that ultraviolet radiation was the strongest contributor to lifespan (41.1%) and average age (37.8%), while temperature seasonality most strongly influenced SVL (43.7%) and age at sexual maturity (40.8%). Annual precipitation was the second most important contributor for age at sexual maturity (31.8%), but had only minor effects on lifespan (16.4%) and SVL (8.1%), ranking third for average age (18.2%). Annual mean temperature showed relatively lower contribution rates across traits (5.5–20.2%), with its highest influence on lifespan (20.2%).

### Prediction of future age and SVL

**Fig. 3:**
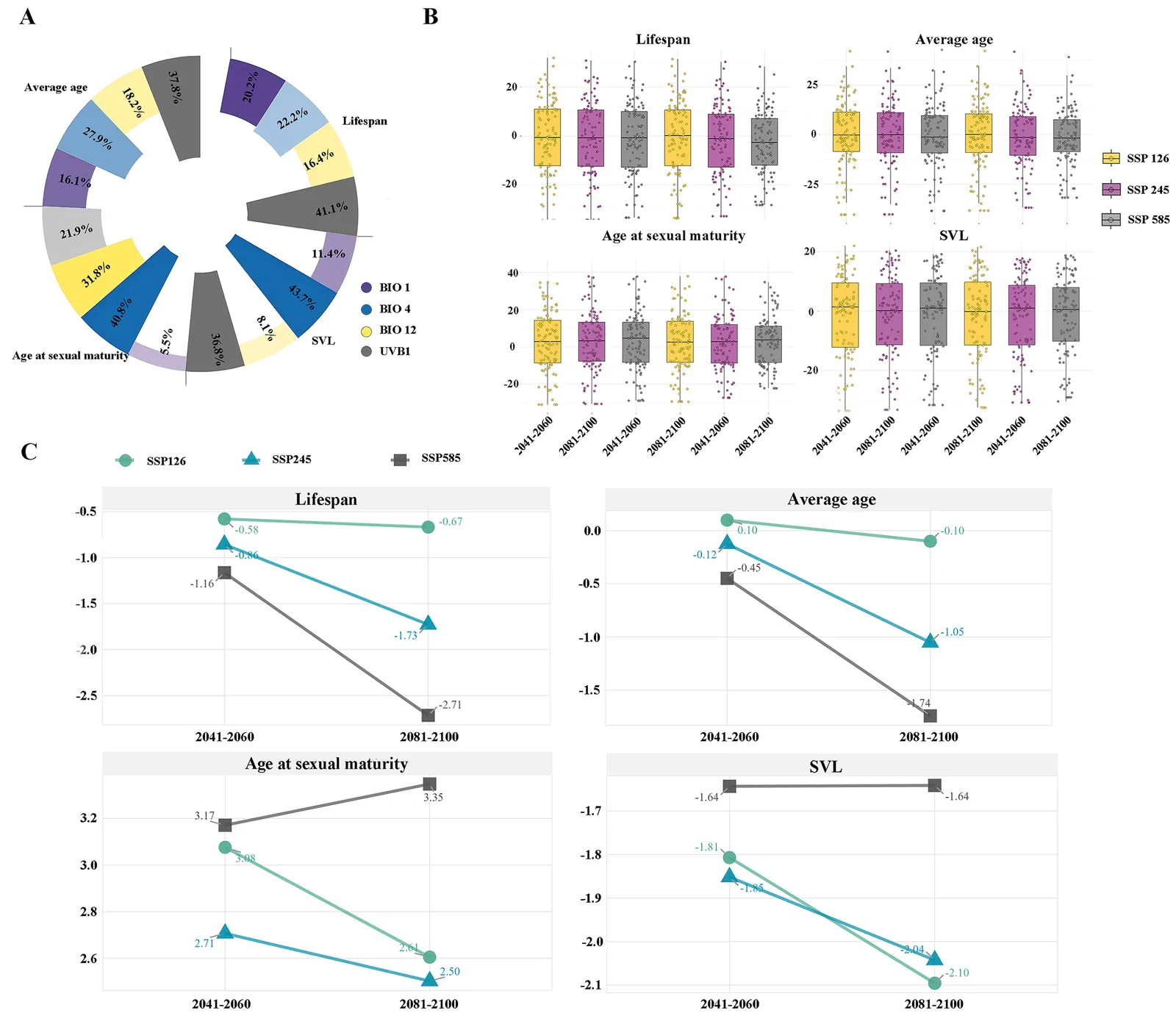
Independent contributions of climatic factors to anuran age structure and max SVL, along with future projections under climate change scenarios. **A** Hierarchical partitioning results showing percentage independent contributions of each climatic variable to age-related traits and SVL. Colors represent climatic predictors: purple = BIO1 (annual mean temperature), blue = BIO4 (temperature seasonality), yellow = BIO12 (annual precipitation), gray = UVB1 (annual mean UV-B). **B** Projected values of age traits and SVL under different Shared Socioeconomic Pathways (SSPs) and time periods: 2041–2060 and 2081–2100. Colors indicate emission scenarios: yellow = SSP126 (low), purple = SSP245 (intermediate), gray = SSP585 (high). **C** Mean projected changes in age structure and SVL from the mid- to late-century. Colors correspond to SSP scenarios: green = SSP126, blue = SSP245, gray = SSP585

Using the random forest modeling framework, we projected changes in age structure and max SVL across three Shared Socioeconomic Pathways (SSP126, SSP245, and SSP585) for the periods 2041–2060 and 2081–2100 (Fig. 3B). An overall decline in lifespan was projected across all scenarios, with the most pronounced reduction occurring under the high-emission scenario (SSP585). By 2041–2060, lifespan was expected to decrease by 1.16% under SSP585, intensifying to 2.71% by the end of the century (2081–2100). Similarly, average age showed an overall declining tendency, ranging from a slight increase (+ 0.10%) under SSP126 to a decline of 0.45% under SSP585 during 2041–2060. By 2081–2100, these declines were projected to reach 0.10% under SSP126 and 1.74% under SSP585. In contrast, age at sexual maturity exhibited a widespread delay across scenarios. The most substantial delay was projected under SSP585, increasing by 3.17% during 2041–2060 and reaching 3.35% by 2081–2100, exceeding delays under other SSPs. Max SVL was predicted to decline across most scenarios, although the magnitude of change varied among SSPs. During 2041–2060, reductions ranged from 1.64% to 1.85%, while by 2081–2100, declines deepened under SSP126 (− 2.10%) and SSP245 (− 2.04%), whereas values under SSP585 remained relatively stable at approximately − 1.64% (Fig. 3C).

### Prediction of age and SVL across habitats

**Fig. 4:**
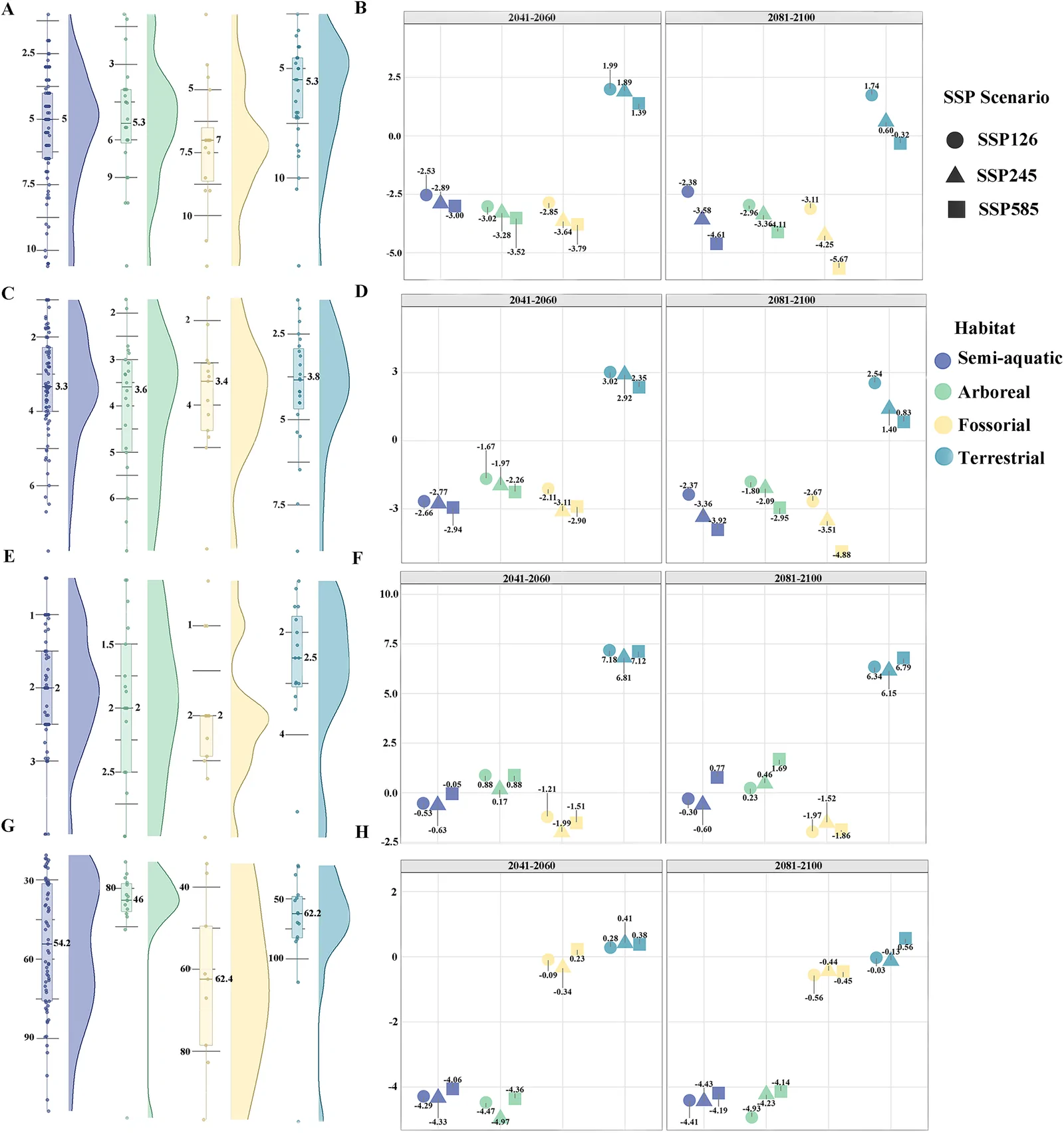
Current and future variation in age structure and max SVL across habitat types, including lifespan (**A**,** B**), average age (**C**,** D**), age at sexual maturity (**E**,** F**), and SVL (**G**,** H**). Left panels (**A**,** C**,** E**,** G**) show current trait values across habitats; right panels (**B**,** D**,** F**,** H**) depict projected future changes under three SSP scenarios. Shapes represent climate scenarios: circle indicates SSP126, triangle indicates SSP245, square indicates SSP585. Colors indicate habitat types: purple indicates semi-aquatic, green indicates arboreal, yellow indicates fossorial, blue indicates terrestrial

Under the current conditions, fossorial species exhibited the longest mean lifespan (7 years), followed by arboreal and terrestrial species (both 5.3 years), with semi-aquatic species having the shortest lifespan (5 years; Fig. 4A). Future projections indicated a general decline in mean lifespan across semi-aquatic, arboreal and fossorial species (Fig. 4B). The most substantial reduction occurred in fossorial species under the SSP585 scenario during 2081–2100, with a relative decrease of 5.67%. Arboreal and semi-aquatic species also experienced pronounced lifespan declines under the same scenario, decreasing by 4.11% and 4.61%. In contrast, terrestrial species exhibited a slight relative increase in lifespan under low-emission scenarios, peaking at + 1.99% during 2041–2060 under SSP126. However, this trend reversed under high-emission conditions, resulting in a small decline of 0.32% by the end of the century under SSP585.

Current average age values were highest in terrestrial species (3.8 years), followed by arboreal (3.6 years), fossorial (3.4 years), and semi-aquatic species (3.3 years; Fig. 4C). Projections suggested widespread declines in average age across all habitat types except terrestrial species (Fig. 4D). The most severe reductions were observed under SSP585 during 2081–2100, with fossorial species declining by 4.88%, semi-aquatic species by 3.92%, and arboreal species by 2.95%. Terrestrial species showed a consistent relative increase in average age under low-emission scenarios, reaching + 3.02% during 2041–2060 under SSP126, although the magnitude of increase diminished under higher emission pathways, reaching + 0.83% under SSP585 by 2081–2100.

At present, semi-aquatic, arboreal, and fossorial species exhibited similar age at sexual maturity (2 years), while terrestrial species matured later (2.5 years; Fig. 4E). Future projections revealed divergent trends among habitat groups (Fig. 4F). Terrestrial species exhibited the strongest relative delay across all scenarios, with increases ranging from 6.15% to 7.18%. Arboreal species exhibited delayed maturation, reaching a maximum increase of 1.69% under SSP585 during 2081–2100. Semi-aquatic species generally showed slight advances in maturation under low- and intermediate-emission scenarios, but shifted toward delayed maturation under high-emission conditions, with a relative increase of 0.77% under SSP585 during 2081–2100. Fossorial species predominantly exhibited early sexual maturity across all scenarios, with the greatest reduction (− 1.99%) occurring under SSP245 during 2041–2060.

Fossorial species currently possessed the largest body size (max SVL 62.4 mm), followed by terrestrial (62.2 mm), semi-aquatic (54.2 mm), and arboreal species (46.0 mm; Fig. 4G). Projected changes in max SVL indicated pronounced reductions in semi-aquatic and arboreal species across all scenarios (Fig. 4H). Arboreal species exhibited the greatest decline under SSP245 during 2041–2060 (− 4.97%), while semi-aquatic species showed the largest reduction under SSP245 during 2081–2100 (− 4.43%). Fossorial species exhibited minimal change, generally remaining within ± 0.6% across scenarios. Terrestrial species showed a consistent slight increase in body size during mid-century (0.28–0.41%), followed by a modest decline toward the end of the century (− 0.03 to − 0.13%).

## Discussion

Our study systematically identifies the key drivers underlying age variation in anurans and suggests how complex interactions among these factors collectively structure demographic patterns. We also uncover sex-specific strategies in survival and reproduction, highlighting the potential role of environmental variability on age structure. By incorporating change projections, we further evaluate potential future shifts in anuran age structure and quantify the relative contributions of major climate drivers to these projected changes.

### Influence of climate change on age structure

Temperature seasonality has emerged as a key factor influencing body size variation in anurans [45], and interactively regulating age at sexual maturity in conjunction with annual precipitation. Elevated temperature seasonality forces individuals to reallocate energy toward stress responses and growth, which may contribute to delayed sexual maturity [75]. Conversely, lower temperatures were positively associated with average age, consistent with reduced winter metabolic activity and oxidative damage and extend the energy accumulation period necessary for reaching reproductive threshold [28, 76]. UV-B radiation is a significant negative predictor of lifespan, with ozone depletion and ongoing climate warming may increase both the strength and variability of UV-B exposure across many regions [77]. This increased exposure can accelerate epidermal cell degeneration and promote premature aging, ultimately reducing both average age and lifespan [78]. During the breeding season, energy distribution is often tied to reproductive activities, such as the production of testosterone and corticosterone, which tend to reduce investment in immune responses [79]. We hypothesize that males with larger body size and greater energy reserves are better equipped to meet these high energy demands and enhance their antioxidant capacity, effectively offsetting the oxidative damage caused by UV-B. In contrast, females, due to the energy demands of reproduction, may be more vulnerable. Their reproductive efforts increase energy expenditure, diminishing their ability to mitigate UV-B damage [80]. Furthermore, temperature and body size interact synergistically to negatively affect the average age of females. High-temperature conditions impair energy storage capacity and reduce survival duration, underscoring a dual vulnerability in physiological tolerance and resource allocation [81, 82]. Precipitation, however, has a positive synergistic relationship with body size in shaping lifespan, reflecting adaptive energy management strategies [83]. Extreme rainfall events, on the other hand, promote the proliferation of aquatic pathogens, increasing mortality during larval and juvenile stages [84]. Additionally, more frequent flooding degrades habitats, prompting compensatory reproductive efforts that accelerate senescence and reduce average age [85].

Climatic factors interact across multiple biological levels to shape the age structure of anurans. Our analysis revealed that the synergistic effect of annual precipitation and UV-B exposure is associated with increased lifespan, age at sexual maturity, and average age. Regions with higher precipitation offer abundant food resources that support individual physiological maintenance and damage repair, while stable hydrology and dense vegetation indirectly help mitigate UV-B induced damage [28]. In contrast, precipitation instability can exacerbate stress, and when coupled with elevated temperatures, it can reduce average age. Together, temperature and precipitation were associated with activity budgets and energy acquisition, which may constrain growth, reproductive output, and offspring traits. These constraints can favour earlier maturation, potentially contributing to lower population-level average age [86, 87]. Furthermore, we found that the interaction between temperature seasonality and UV-B exposure is associated with increases in both lifespan and average age, suggesting a potential buffering effect. This pattern is consistent with behavioral adaptations, such as shifts in diurnal activity patterns, which may be facilitated by temperature seasonality [56, 88]. Although UV-B and temperature seasonality exhibited a negative interaction that was associated with reduced age at sexual maturity, temperature seasonality remained a core buffering factor within this synergistic framework. This is largely because high-temperature seasons were associated with shorter growth periods, favoring accelerated development and earlier maturation, which may enable greater reproductive success [39].

### Influence of geographical gradient on age structure

This study elucidates the effects of elevation and latitude on age-related traits and snout-vent length (SVL) in anurans. Latitude emerged as a significant positive predictor of lifespan, which we attribute to the buffering capacity of high-latitude environments. For instance, cooler temperatures at higher latitudes are associated with reduced metabolic rates, decreased oxidative stress, and extended hibernation durations—factors all contributing to longer lifespans [89]. Our findings also reveal a synergistic effect between latitude and SVL, which was associated with later sexual maturity. Species in higher latitudes prolong their growth period to accumulate sufficient energy reserves for reproduction, resulting in increases in both average population age and lifespan [67, 90]. Additionally, reduced predation pressure and shorter active seasons in these regions promote energy allocation toward self-maintenance and overwintering preparedness, further elevating both average age and lifespan [86]. Similarly, elevation interacts positively with body size, driving high-elevation taxa toward larger body sizes. This shift reduces extrinsic mortality and supports longer lifespans [91]. However, under extreme conditions, antagonistic effects between latitude and elevation become evident. In high-latitude regions, the longevity benefits of low temperatures are often counteracted by oxidative damage caused by high-elevation hypoxia and intense UV-B [27, 81]. These adverse effects are particularly pronounced in females, where prolonged oocyte maturation under cold stress, hypoxia-induced mitochondrial dysfunction, and accelerated oxidative damage accumulation collectively lead to shorter lifespans compared to males [92].

### Influence of habitat types on age structure

Our study shows that under current climate conditions, broad habitat types do not significantly explain variations in age structure. However, in the context of intensifying climate change, different habitat groups exhibit marked differences in their responses to environmental pressures [93]. Previous research suggests that microhabitat conditions play a more critical role in buffering against extreme temperatures and mitigating climate impacts [94]. Projections under future climate scenarios indicate that semi-aquatic and arboreal species show pronounced shifts in age structure, including reduced lifespan and accelerated maturation, which may reflect heightened vulnerability to climate change. Rising temperatures are accelerating the loss and desiccation of wetlands, potentially rendering many anuran populations maladapted [39, 95]. Arboreal species, which often exhibit delayed maturation due to high energy accumulation requirements, rely heavily on forest canopies for shelter, moisture, prey, and protection from predators [96]. However, intensified climate change, especially extreme drought, is degrading these arboreal and semi-aquatic habitats. This disruption directly impacts resource availability, which is necessary for delayed maturation, and may force premature reproduction under energy-limited conditions [97]. These pressures contribute to continued reduction in average body size [98], and altered age structure in arboreal and semi-aquatic anurans, which may carry implications for demographic fitness [35]. Drought conditions also compel arboreal species to descend in search of water, increasing competitive interactions with terrestrial and fossorial species [99]. Studies in Papua New Guinea have shown that such vertical competition disproportionately affects late-maturing species [100], thereby potentially elevating their vulnerability to drought event [101]. Thus, these groups should be prioritized for assessment and monitoring to confirm their risks under climate change scenarios. Furthermore, climate-induced changes in precipitation patterns are disrupting hydrological cycles, leading to increased egg mortality for all anurans that rely on aquatic environments for reproduction. This includes not only semi-aquatic species but also terrestrial, arboreal and fossorial species [40]. Fossorial species are highly sensitive to habitat degradation, typically responding with shortened lifespans, reduced average age, earlier sexual maturity, and increased body size. Drought is recognized as one of the most severe threats to fossorial species [94]. Drying soils driven by higher temperatures and reduced precipitation force these species to burrow deeper to avoid desiccation and mortality [82]. In contrast, terrestrial species show stronger short-term resilience but exhibit time-dependent vulnerability under long-term high-emission scenarios, with their adaptive advantages gradually diminishing over time.

### Influence of body size on age structure

Our results highlight the pivotal role of body size in shaping age structures in anurans, a pattern consistent with broader tetrapod lineages [28, 102–104]. Specifically, body size exerts an indirect positive effect on both average age and lifespan by delaying sexual maturation. Compared with smaller species, larger anurans experience lower extrinsic mortality rates, due to reduced vulnerability to temporary food shortages, climatic fluctuations, extreme weather events, and potentially lower predation pressure. These factors collectively contribute to extended longevity [35]. Moreover, larger body size is associated with slower metabolic rates, resulting in slower growth. This physiological trait not only extends the time required to reach sexual maturity but may also enhance lifespan by reducing the accumulation of oxidative damage [105]. The influence of body size on lifespan, average age, and age at sexual maturity is particularly pronounced in male anurans. In the context of ongoing climate change, a global decline in average body size across numerous animal taxa has been documented [102, 106], a trend also corroborated by our model outcomes. Warming temperatures accelerate growth and development rates, but ultimately reduce maximum adult body size, leading to earlier sexual maturation at smaller sizes [107].

### Future prediction of age structure

Future climate change, particularly under high-emission scenarios, is linked to pronounced shifts in age structure, while potentially altering thermoregulatory patterns historically tied to winter metabolic depression, and intensifying multiple environmental stressors, including extreme weather events and heightened ultraviolet radiation [108]. Persistently rising mean annual temperatures increase metabolic demands during overwintering, leading to accelerated depletion of energy reserves [109]. This depletion may compromise individual physiological condition and reduce overwinter survival rates [110], becoming a critical driver of altered age structures. Concurrently, more frequent extreme weather events—such as unseasonal spring warming—raise the risk of cold exposure, impairing thermoregulatory capacity with potential consequences for reproductive output [111]. Irregular precipitation patterns can also induce premature reproduction in immature individuals, triggering potential energy allocation trade-offs and elevated oxidative stress [87]. These combined pressures force organisms to divert limited energy resources toward immediate growth and reproductive demands at the expense of long-term cellular maintenance and repair [76]. This pattern parallels reduced investment in maintenance physiology, a dynamic that may contribute to the observed reduction in individual lifespans. The reallocation of energy resources also helps explain the ongoing decline in average body size, signaling a loss of the protective buffering historically provided by temperature seasonality. While climate warming generally accelerates life-history trajectories, severe energy constraints hinder individuals from reaching essential reproductive thresholds, resulting in significant delays in sexual maturation [112]. Under high-emission scenarios, this delay becomes more pronounced, highlighting the antagonistic interaction between thermally accelerated development and energy-limited postponement of maturation. In summary, climate change, particularly under high-emission scenarios, is tied to substantial shifts in amphibian age structures, characterized by younger population age structures, reduced body size, and delayed reproductive maturation.

## Conclusions

In the context of global climate change, this study quantitatively analyzed the dynamic relationships between environmental factors and age structure in anurans, and systematically projected potential changes in anuran age structure under future climate scenarios. Our results indicate that annual mean temperature, annual precipitation, temperature seasonality, ultraviolet radiation, latitude, elevation, and SVL are linked to age-structure parameters through direct, indirect, and synergistic pathways, with notable sex-specific differences. Under future climate conditions, lifespan, average age, and SVL are projected to decline across various habitat categories, indicating a broad trend toward smaller body size and younger age structures in anuran species. By quantifying the dynamic links between environmental stress and life-history traits, this study provides critical insights into the mechanisms underlying global amphibian declines. Furthermore, it offers a scientific foundation—grounded in age-structure analysis—for developing targeted conservation strategies, thereby supporting efforts to mitigate ongoing species declines. Given the escalating impacts of climate change on anuran adaptability, future research should prioritize understanding how extreme climate events influence life-history trajectories and demographic resilience. 

## Supplementary Information

Below is the link to the electronic supplementary material.

Supplementary Material 1

## Data Availability

The datasets supporting the conclusions of this article are publicly available in the Figshare repository at https://doi.org/10.6084/m9.figshare.30928787.
