# Supplementary material for: Uncovering the drivers of anuran age structure
Source: Front Zool. 2026 Aug 27;23:33. doi: 10.1186/s12983-026-00630-5 (PMC13599300; doi:10.1186/s12983-026-00630-5)
Supplement: Supplementary file 1 [file 12983_2026_630_MOESM1_ESM.docx]

**TableS1** Full model set with geographical variables (elevation and latitude), including all interaction terms among explanatory variables.

| **Data_set** | **Parameter** | **Estimate±SE** | **T value** | ***p*** | **R^2^** | **95%CI** | **F** | **λ** | **Model *p*** |
| --- | --- | --- | --- | --- | --- | --- | --- | --- | --- |
| **Lifespan** |  |  |  |  |  |  |  |  |  |
| Total | (Intercept) | -1.513±1.618 | -0.935 | 0.354 | 0.682 | (NA,0.376) | 6.429 | 0.000 | <0.001 |
|  | Elevation | 0.197±0.410 | 0.481 | 0.633 |  |  |  |  |  |
|  | Latitude | 1.617±1.024 | 1.580 | 0.120 |  |  |  |  |  |
|  | SVL max | 0.060±0.927 | 0.065 | 0.949 |  |  |  |  |  |
|  | Habitat Arboreal | -0.881±1.008 | -0.874 | 0.386 |  |  |  |  |  |
|  | Habitat Fossorial | -2.434±1.381 | -1.763 | 0.084 |  |  |  |  |  |
|  | Habitat Terrestrial | 0.073±0.546 | 0.133 | 0.894 |  |  |  |  |  |
|  | Elevation: Latitude | -0.324±0.252 | -1.284 | 0.205 |  |  |  |  |  |
|  | Elevation:SVL max | 0.211±0.212 | 0.992 | 0.325 |  |  |  |  |  |
|  | Latitude:SVL max | -0.192±0.441 | -0.435 | 0.665 |  |  |  |  |  |
|  | Elevation:Habitat Arboreal | 0.021±0.114 | 0.182 | 0.856 |  |  |  |  |  |
|  | Elevation:Habitat Fossorial | -0.046±0.113 | -0.408 | 0.685 |  |  |  |  |  |
|  | Elevation:Habitat Terrestrial | -0.029±0.086 | -0.340 | 0.735 |  |  |  |  |  |
|  | Latitude:Habitat Arboreal | -0.015±0.213 | -0.070 | 0.945 |  |  |  |  |  |
|  | Latitude:Habitat Fossorial | 0.750±0.556 | 1.347 | 0.183 |  |  |  |  |  |
|  | Latitude:Habitat Terrestrial | -0.273±0.285 | -0.961 | 0.341 |  |  |  |  |  |
|  | SVL max:Habitat Arboreal | 0.513±0.706 | 0.727 | 0.471 |  |  |  |  |  |
|  | SVL max:Habitat Fossorial | 0.822±0.604 | 1.361 | 0.179 |  |  |  |  |  |
|  | SVL max:Habitat Terrestrial | 0.251±0.246 | 1.020 | 0.312 |  |  |  |  |  |
| Males | Intercept | -1.059±1.719 | -0.616 | 0.540 | 0.648 | (NA,0.523) | 5.428 | 0 | <0.001 |
|  | Elevation | 0.295±0.430 | 0.686 | 0.496 |  |  |  |  |  |
|  | Latitude | 1.451±1.090 | 1.331 | 0.189 |  |  |  |  |  |
|  | SVL max | -0.523±1.018 | -0.513 | 0.610 |  |  |  |  |  |
|  | Habitat Arboreal | -0.927±1.059 | -0.875 | 0.385 |  |  |  |  |  |
|  | Habitat Fossorial | -2.764±1.332 | -2.074 | 0.043 |  |  |  |  |  |
|  | Habitat Terrestrial | 0.241±0.603 | 0.399 | 0.691 |  |  |  |  |  |
|  | Elevation:Latitude | -0.402±0.266 | -1.515 | 0.136 |  |  |  |  |  |
|  | Elevation:SVL max | 0.233±0.235 | 0.992 | 0.326 |  |  |  |  |  |
|  | Elevation:Habitat Arboreal | 0.036±0.117 | 0.311 | 0.757 |  |  |  |  |  |
|  | Elevation:Habitat Fossorial | -0.007±0.113 | -0.058 | 0.954 |  |  |  |  |  |
|  | Elevation:Habitat Terrestrial | -0.058±0.086 | -0.675 | 0.502 |  |  |  |  |  |
|  | Latitude:SVL max | 0.098±0.481 | 0.203 | 0.840 |  |  |  |  |  |
|  | Latitude:Habitat Arboreal | 0.028±0.207 | 0.136 | 0.892 |  |  |  |  |  |
|  | Latitude:Habitat Fossorial | 0.473±0.582 | 0.812 | 0.420 |  |  |  |  |  |
|  | Latitude:Habitat Terrestrial | -0.400±0.302 | -1.322 | 0.192 |  |  |  |  |  |
|  | SVL max:Habitat Arboreal | 0.477±0.710 | 0.672 | 0.505 |  |  |  |  |  |
|  | SVL max:Habitat Fossorial | 1.204±0.585 | 2.058 | 0.045 |  |  |  |  |  |
|  | SVL max:Habitat Terrestrial | 0.310±0.296 | 1.047 | 0.300 |  |  |  |  |  |
| Females | Intercept | 1.823±2.056 | 0.887 | 0.380 | 0.685 | (NA,0.182) | 3.979 | 0.000 | <0.001 |
|  | Elevation | -1.041±0.547 | -1.901 | 0.064 |  |  |  |  |  |
|  | Latitude | 0.633±1.208 | 0.524 | 0.603 |  |  |  |  |  |
|  | SVL max | -0.784±0.995 | -0.789 | 0.434 |  |  |  |  |  |
|  | Habitat Arboreal | -1.093±1.125 | -0.972 | 0.336 |  |  |  |  |  |
|  | Habitat Fossorial | -1.268±1.546 | -0.820 | 0.416 |  |  |  |  |  |
|  | Habitat Terrestrial | -0.628±0.601 | -1.044 | 0.302 |  |  |  |  |  |
|  | Elevation:Latitude | 0.015±0.279 | 0.052 | 0.959 |  |  |  |  |  |
|  | Elevation:SVL max | 0.626±0.243 | 2.578 | 0.013 |  |  |  |  |  |
|  | Latitude:SVL max | -0.338±0.495 | -0.682 | 0.499 |  |  |  |  |  |
|  | Habitat Arboreal:Elevation | 0.072±0.211 | 0.339 | 0.736 |  |  |  |  |  |
|  | Habitat Fossorial:Elevation | -0.270±0.132 | -2.039 | 0.047 |  |  |  |  |  |
|  | Habitat Terrestrial:Elevation | 0.173±0.104 | 1.655 | 0.105 |  |  |  |  |  |
|  | Habitat Arboreal:Latitude | 0.673±1.321 | 0.509 | 0.613 |  |  |  |  |  |
|  | Habitat Fossorial:Latitude | 1.323±0.575 | 2.300 | 0.026 |  |  |  |  |  |
|  | Habitat Terrestrial:Latitude | 0.004±0.304 | 0.012 | 0.991 |  |  |  |  |  |
|  | Habitat Arboreal:SVL max | -0.046±1.803 | -0.025 | 0.980 |  |  |  |  |  |
|  | Habitat Fossorial:SVL max | 0.007±0.702 | 0.011 | 0.992 |  |  |  |  |  |
|  | Habitat Terrestrial:SVL max | 0.079±0.239 | 0.328 | 0.744 |  |  |  |  |  |
| **Age at Sexual Maturity** | | | | | | | | | |
| Total | Intercept | 2.700±1.940 | 1.392 | 0.170 | 0.507 | (0.368,0.912) | 2.796 | 0.725 | 0.002 |
|  | Elevation | -0.716±0.515 | -1.392 | 0.170 |  |  |  |  |  |
|  | Latitude | -0.826±1.128 | -0.732 | 0.467 |  |  |  |  |  |
|  | SVL max | -1.406±1.119 | -1.256 | 0.215 |  |  |  |  |  |
|  | Habitat Arboreal | 0.544±0.975 | 0.558 | 0.580 |  |  |  |  |  |
|  | Habitat Fossorial | -2.724±1.441 | -1.890 | 0.065 |  |  |  |  |  |
|  | Habitat Terrestrial | -0.682±0.654 | -1.043 | 0.302 |  |  |  |  |  |
|  | Elevation:Latitude | 0.150±0.270 | 0.556 | 0.581 |  |  |  |  |  |
|  | Elevation:SV Lmax | 0.302±0.285 | 1.061 | 0.294 |  |  |  |  |  |
|  | Latitude:SVL max | 0.051±0.098 | 0.519 | 0.606 |  |  |  |  |  |
|  | Elevation:Habitat Arboreal | -0.084±0.127 | -0.661 | 0.512 |  |  |  |  |  |
|  | Elevation:Habitat Fossorial | 0.095±0.103 | 0.920 | 0.362 |  |  |  |  |  |
|  | Elevation:Habitat Terrestrial | 0.489±0.473 | 1.033 | 0.307 |  |  |  |  |  |
|  | Latitude:Habitat Arboreal | -0.022±0.193 | -0.114 | 0.910 |  |  |  |  |  |
|  | Latitude:Habitat Fossorial | 0.060±0.692 | 0.086 | 0.932 |  |  |  |  |  |
|  | Latitude:Habitat Terrestrial | 0.011±0.270 | 0.042 | 0.967 |  |  |  |  |  |
|  | SVL max:Habitat Arboreal | -0.371±0.689 | -0.538 | 0.593 |  |  |  |  |  |
|  | SVL max:Habitat Fossorial | 1.431±0.646 | 2.215 | 0.031 |  |  |  |  |  |
|  | SVL max:Habitat Terrestrial | 0.221±0.251 | 0.882 | 0.382 |  |  |  |  |  |
| Males | Intercept | 3.435±2.124 | 1.617 | 0.113 | 0.401 | (0.237,0.882) | 1.638 | 0.670 | 0.092 |
|  | Elevation | -1.005±0.595 | -1.689 | 0.098 |  |  |  |  |  |
|  | Latitude | -1.016±1.167 | -0.871 | 0.389 |  |  |  |  |  |
|  | SVL | -1.406±1.340 | -1.049 | 0.300 |  |  |  |  |  |
|  | Habitat Arboreal | 0.342±1.049 | 0.326 | 0.746 |  |  |  |  |  |
|  | Habitat Fossorial | -2.650±2.790 | -0.950 | 0.347 |  |  |  |  |  |
|  | Habitat Terrestrial | -1.103±0.697 | -1.582 | 0.121 |  |  |  |  |  |
|  | Elevation:Latitude | 0.257±0.299 | 0.859 | 0.395 |  |  |  |  |  |
|  | Elevation:SVL | 0.385±0.362 | 1.063 | 0.294 |  |  |  |  |  |
|  | Elevation:Habitat Arboreal | 0.043±0.101 | 0.422 | 0.675 |  |  |  |  |  |
|  | Elevation:Habitat Fossorial | -0.165±0.122 | -1.349 | 0.184 |  |  |  |  |  |
|  | Elevation:Habitat Terrestrial | 0.165±0.107 | 1.532 | 0.133 |  |  |  |  |  |
|  | Latitude:SVL | 0.288±0.510 | 0.565 | 0.575 |  |  |  |  |  |
|  | Latitude:Habitat Arboreal | 0.127±0.208 | 0.609 | 0.546 |  |  |  |  |  |
|  | Latitude:Habitat Fossorial | 0.474±0.918 | 0.516 | 0.608 |  |  |  |  |  |
|  | Latitude:Habitat Terrestrial | 0.122±0.291 | 0.418 | 0.678 |  |  |  |  |  |
|  | SVL:Habitat Arboreal | -0.367±0.717 | -0.512 | 0.612 |  |  |  |  |  |
|  | SVL:Habitat Fossorial | 1.196±1.050 | 1.139 | 0.261 |  |  |  |  |  |
|  | SVL:Habitat Terrestrial | 0.249±0.295 | 0.845 | 0.403 |  |  |  |  |  |
| Females | Intercept | 1.671±2.083 | 0.802 | 0.427 | 0.402 | (0.287,0.896) | 1.683 | 0.693 | 0.079 |
|  | Elevation | -0.336±0.565 | -0.595 | 0.555 |  |  |  |  |  |
|  | Latitude | -0.714±1.100 | -0.649 | 0.520 |  |  |  |  |  |
|  | SVL | -0.934±1.101 | -0.848 | 0.401 |  |  |  |  |  |
|  | Habitat Arboreal | 0.612±1.151 | 0.532 | 0.597 |  |  |  |  |  |
|  | Habitat Fossorial | -1.428±1.570 | -0.909 | 0.368 |  |  |  |  |  |
|  | Habitat Terrestrial | -0.356±0.714 | -0.499 | 0.620 |  |  |  |  |  |
|  | Elevation:Latitude | 0.030±0.245 | 0.122 | 0.903 |  |  |  |  |  |
|  | Elevation:SVL | 0.166±0.282 | 0.590 | 0.558 |  |  |  |  |  |
|  | Elevation:Habitat Arboreal | 0.089±0.128 | 0.696 | 0.490 |  |  |  |  |  |
|  | Elevation:Habitat Fossorial | -0.010±0.141 | -0.068 | 0.946 |  |  |  |  |  |
|  | Elevation:Habitat Terrestrial | 0.064±0.101 | 0.639 | 0.526 |  |  |  |  |  |
|  | Latitude:SVL | 0.549±0.500 | 1.099 | 0.278 |  |  |  |  |  |
|  | Latitude:Habitat Arboreal | 0.288±0.287 | 1.004 | 0.321 |  |  |  |  |  |
|  | Latitude:Habitat Fossorial | -0.510±0.717 | -0.712 | 0.480 |  |  |  |  |  |
|  | Latitude:Habitat Terrestrial | 0.092±0.288 | 0.319 | 0.751 |  |  |  |  |  |
|  | SVL:Habitat Arboreal | -0.740±0.947 | -0.782 | 0.438 |  |  |  |  |  |
|  | SVL:Habitat Fossorial | 1.086±0.697 | 1.559 | 0.126 |  |  |  |  |  |
|  | SVL:Habitat Terrestrial | 0.018±0.274 | 0.066 | 0.948 |  |  |  |  |  |
| **Average age** |  |  |  |  |  |  |  |  |  |
| Total | Intercept | 0.886±1.517 | 0.584 | 0.561 | 0.014 | (0.374,0.930) | 2.661 | 0.772 | 0.002 |
|  | Elevation | 0.224±0.337 | 0.664 | 0.509 |  |  |  |  |  |
|  | Latitude | -0.847±0.980 | -0.864 | 0.391 |  |  |  |  |  |
|  | SVL | -0.978±0.916 | -1.067 | 0.290 |  |  |  |  |  |
|  | Habitat Arboreal | -0.041±0.690 | -0.059 | 0.953 |  |  |  |  |  |
|  | Habitat Fossorial | -0.849±1.371 | -0.619 | 0.538 |  |  |  |  |  |
|  | Habitat Terrestrial | -0.132±0.579 | -0.227 | 0.821 |  |  |  |  |  |
|  | Elevation:Latitude | -0.116±0.221 | -0.525 | 0.602 |  |  |  |  |  |
|  | Elevation:SVL | -0.029±0.191 | -0.154 | 0.878 |  |  |  |  |  |
|  | Elevation:Habitat Arboreal | 0.069±0.088 | 0.781 | 0.438 |  |  |  |  |  |
|  | Elevation:Habitat Fossorial | 0.004±0.087 | 0.049 | 0.961 |  |  |  |  |  |
|  | Elevation:Habitat Terrestrial | 0.060±0.073 | 0.818 | 0.416 |  |  |  |  |  |
|  | Latitude:SVL | 1.046±0.458 | 2.283 | 0.026 |  |  |  |  |  |
|  | Latitude:Habitat Arboreal | 0.140±0.184 | 0.763 | 0.448 |  |  |  |  |  |
|  | Latitude:Habitat Fossorial | -0.184±0.651 | -0.284 | 0.778 |  |  |  |  |  |
|  | Latitude:Habitat Terrestrial | -0.056±0.234 | -0.238 | 0.813 |  |  |  |  |  |
|  | SVL:Habitat Arboreal | -0.208±0.507 | -0.409 | 0.684 |  |  |  |  |  |
|  | SVL:Habitat Fossorial | 0.549±0.551 | 0.995 | 0.323 |  |  |  |  |  |
|  | SVL:Habitat Terrestrial | 0.025±0.249 | 0.101 | 0.920 |  |  |  |  |  |
| Males | Intercept | 1.975±1.758 | 1.123 | 0.266 | 0.493 | (NA,0.754) | 3.346 | 0.418 | <0.001 |
|  | Elevation | -0.333±0.442 | -0.752 | 0.455 |  |  |  |  |  |
|  | Latitude | -0.940±1.105 | -0.851 | 0.398 |  |  |  |  |  |
|  | SVL | -1.159±0.967 | -1.199 | 0.235 |  |  |  |  |  |
|  | Habitat Arboreal | 0.097±0.783 | 0.124 | 0.902 |  |  |  |  |  |
|  | Habitat Fossorial | -1.624±1.351 | -1.202 | 0.234 |  |  |  |  |  |
|  | Habitat Terrestrial | -0.511±0.614 | -0.833 | 0.408 |  |  |  |  |  |
|  | Elevation:Latitude | 0.026±0.263 | 0.100 | 0.921 |  |  |  |  |  |
|  | Elevation:SVL | 0.167±0.215 | 0.779 | 0.439 |  |  |  |  |  |
|  | Elevation:Habitat Arboreal | 0.080±0.104 | 0.765 | 0.447 |  |  |  |  |  |
|  | Elevation:Habitat Fossorial | -0.040±0.105 | -0.377 | 0.707 |  |  |  |  |  |
|  | Elevation:Habitat Terrestrial | 0.138±0.088 | 1.567 | 0.122 |  |  |  |  |  |
|  | Latitude:SVL | 0.787±0.478 | 1.648 | 0.104 |  |  |  |  |  |
|  | Latitude:Habitat Arboreal | 0.203±0.204 | 0.997 | 0.323 |  |  |  |  |  |
|  | Latitude:Habitat Fossorial | 0.282±0.635 | 0.445 | 0.658 |  |  |  |  |  |
|  | Latitude:Habitat Terrestrial | 0.044±0.283 | 0.155 | 0.877 |  |  |  |  |  |
|  | SVL:Habitat Arboreal | -0.360±0.570 | -0.632 | 0.530 |  |  |  |  |  |
|  | SVL:Habitat Fossorial | 0.685±0.541 | 1.266 | 0.210 |  |  |  |  |  |
|  | SVL:Habitat Terrestrial | 0.033±0.271 | 0.123 | 0.903 |  |  |  |  |  |
| Females | Intercept | 1.506±1.841 | 0.818 | 0.417 | 0.457 | (0.046,0.940) | 2.711 | 0.655 | 0.002 |
|  | Elevation | -0.140±0.450 | -0.312 | 0.757 |  |  |  |  |  |
|  | Latitude | -0.940±1.085 | -0.867 | 0.390 |  |  |  |  |  |
|  | SVL | -1.041±1.035 | -1.006 | 0.318 |  |  |  |  |  |
|  | Habitat Arboreal | 0.048±0.762 | 0.063 | 0.950 |  |  |  |  |  |
|  | Habitat Fossorial | -0.555±1.514 | -0.366 | 0.715 |  |  |  |  |  |
|  | Habitat Terrestrial | -0.276±0.635 | -0.435 | 0.666 |  |  |  |  |  |
|  | Elevation:Latitude | -0.016±0.241 | -0.067 | 0.947 |  |  |  |  |  |
|  | Elevation:SVL | 0.101±0.226 | 0.444 | 0.659 |  |  |  |  |  |
|  | Elevation:Habitat Arboreal | 0.086±0.103 | 0.828 | 0.411 |  |  |  |  |  |
|  | Elevation:Habitat Fossorial | -0.046±0.099 | -0.467 | 0.643 |  |  |  |  |  |
|  | Elevation:Habitat Terrestrial | 0.147±0.086 | 1.707 | 0.093 |  |  |  |  |  |
|  | Latitude:SVL | 0.899±0.505 | 1.780 | 0.080 |  |  |  |  |  |
|  | Latitude:Habitat Arboreal | 0.197±0.216 | 0.912 | 0.365 |  |  |  |  |  |
|  | Latitude:Habitat Fossorial | 0.020±0.658 | 0.031 | 0.975 |  |  |  |  |  |
|  | Latitude:Habitat Terrestrial | -0.056±0.255 | -0.219 | 0.828 |  |  |  |  |  |
|  | SVL:Habitat Arboreal | -0.343±0.572 | -0.599 | 0.551 |  |  |  |  |  |
|  | SVL:Habitat Fossorial | 0.290±0.648 | 0.448 | 0.656 |  |  |  |  |  |
|  | SVL:Habitat Terrestrial | -0.041±0.252 | -0.163 | 0.871 |  |  |  |  |  |

Notes: CI, confidence interval; SVL, snout–vent length. The *p*-value indicates the level of significance of phylogenetic signals

**TableS2** Full model set with climatic variables, including all interaction terms among explanatory variables.

| **Data_set** | **Parameter** | **Estimate±SE** | **T value** | ***p*** | **R^2^** | **95%CI** | **F** | **λ** | **Model *p*** |
| --- | --- | --- | --- | --- | --- | --- | --- | --- | --- |
| **Lifespan** |  |  |  |  |  |  |  |  |  |
| Total | Intercept | 43.514±33.744 | 1.290 | 0.204 | 0.711 | (NA,0.226) | 3.058 | 0.000 | <0.001 |
|  | Bio1 | 5.694±9.283 | 0.613 | 0.543 |  |  |  |  |  |
|  | Bio4 | -5.271±5.231 | -1.008 | 0.320 |  |  |  |  |  |
|  | Bio12 | -10.967±6.996 | -1.568 | 0.125 |  |  |  |  |  |
|  | UVB1 | -13.500±9.012 | -1.498 | 0.142 |  |  |  |  |  |
|  | SVL max | 1.857±10.586 | 0.175 | 0.862 |  |  |  |  |  |
|  | Habitat Arboreal | 2.861±4.524 | 0.632 | 0.531 |  |  |  |  |  |
|  | Habitat Fossorial | -3.683±12.020 | -0.306 | 0.761 |  |  |  |  |  |
|  | Habitat Terrestrial | -2.808±3.579 | -0.785 | 0.437 |  |  |  |  |  |
|  | Bio1:Bio4 | -0.098±0.906 | -0.108 | 0.914 |  |  |  |  |  |
|  | Bio1:Bio12 | -0.870±1.142 | -0.762 | 0.451 |  |  |  |  |  |
|  | Bio1:UVB1 | -0.375±1.564 | -0.240 | 0.812 |  |  |  |  |  |
|  | Bio1:SVL max | -0.902±0.838 | -1.076 | 0.288 |  |  |  |  |  |
|  | Bio4:Bio12 | 0.038±0.506 | 0.075 | 0.940 |  |  |  |  |  |
|  | Bio4:UVB1 | 1.750±1.396 | 1.254 | 0.217 |  |  |  |  |  |
|  | Bio4:SVL max | -0.599±1.003 | -0.597 | 0.554 |  |  |  |  |  |
|  | Bio12:UVB1 | 3.103±1.917 | 1.618 | 0.113 |  |  |  |  |  |
|  | Bio12:SVL max | 0.469±0.734 | 0.638 | 0.527 |  |  |  |  |  |
|  | Uvb1:SVLmax1 | -0.094±2.128 | -0.044 | 0.965 |  |  |  |  |  |
|  | SVL max:Habitat Arboreal | 0.650±0.575 | 1.132 | 0.264 |  |  |  |  |  |
|  | SVL max:Habitat Fossorial | 0.366±0.572 | 0.641 | 0.525 |  |  |  |  |  |
|  | SVL max:Habitat Terrestrial | 0.257±0.275 | 0.938 | 0.354 |  |  |  |  |  |
|  | Habitat Arboreal:Bio1 | -1.075±0.991 | -1.085 | 0.284 |  |  |  |  |  |
|  | Habitat Fossorial:Bio1 | -0.598±0.927 | -0.645 | 0.522 |  |  |  |  |  |
|  | Habitat Terrestrial:Bio1 | -0.185±0.432 | -0.428 | 0.671 |  |  |  |  |  |
|  | Habitat Arboreal:Bio4 | -0.343±0.335 | -1.024 | 0.312 |  |  |  |  |  |
|  | Habitat Fossorial:Bio4 | 0.644±1.739 | 0.370 | 0.713 |  |  |  |  |  |
|  | Habitat Terrestrial:Bio4 | 0.184±0.390 | 0.473 | 0.639 |  |  |  |  |  |
|  | Habitat Arboreal:Bio12 | 0.062±0.363 | 0.171 | 0.865 |  |  |  |  |  |
|  | Habitat Fossorial:Bio12 | -0.569±1.501 | -0.379 | 0.707 |  |  |  |  |  |
|  | Habitat Terrestrial:Bio12 | 0.178±0.289 | 0.617 | 0.541 |  |  |  |  |  |
|  | Habitat Arboreal:UVB1 | -0.566±1.184 | -0.478 | 0.635 |  |  |  |  |  |
|  | Habitat Fossorial:UVB1 | 1.009±2.619 | 0.385 | 0.702 |  |  |  |  |  |
|  | Habitat Terrestrial:UVB1 | 0.433±0.683 | 0.634 | 0.530 |  |  |  |  |  |
| Males | Intercept | 65.031±34.248 | 1.899 | 0.065 | 0.695 | (NA,0.162) | 2.764 | 0 | 0.001 |
|  | Bio1 | 9.500±8.893 | 1.068 | 0.292 |  |  |  |  |  |
|  | Bio4 | -7.924±5.330 | -1.487 | 0.145 |  |  |  |  |  |
|  | Bio12 | -13.884±7.201 | -1.928 | 0.061 |  |  |  |  |  |
|  | UVB1 | -19.739±9.141 | -2.159 | 0.037 |  |  |  |  |  |
|  | SVL max | -3.953±10.106 | -0.391 | 0.698 |  |  |  |  |  |
|  | Habitat Arboreal | 1.881±4.629 | 0.406 | 0.687 |  |  |  |  |  |
|  | Habitat Fossorial | 0.267±11.999 | 0.022 | 0.982 |  |  |  |  |  |
|  | Habitat Terrestrial | -1.967±3.638 | -0.541 | 0.592 |  |  |  |  |  |
|  | Bio1:Bio4 | -0.646±0.929 | -0.695 | 0.491 |  |  |  |  |  |
|  | Bio1:Bio12 | -1.716±1.165 | -1.472 | 0.149 |  |  |  |  |  |
|  | Bio1:UVB1 | -0.554±1.449 | -0.383 | 0.704 |  |  |  |  |  |
|  | SVL max:Bio1 | -0.472±0.959 | -0.492 | 0.626 |  |  |  |  |  |
|  | Bio4:Bio12 | -0.029±0.495 | -0.058 | 0.954 |  |  |  |  |  |
|  | Bio4:UVB1 | 2.399±1.419 | 1.691 | 0.099 |  |  |  |  |  |
|  | SVL max:Bio4 | 0.140±0.961 | 0.145 | 0.885 |  |  |  |  |  |
|  | Bio12:UVB1 | 4.222±1.953 | 2.161 | 0.037 |  |  |  |  |  |
|  | SVL max:Bio12 | 0.597±0.790 | 0.756 | 0.454 |  |  |  |  |  |
|  | SVL max:UVB1 | 0.721±2.026 | 0.356 | 0.724 |  |  |  |  |  |
|  | SVL max:Habitat Arboreal | 0.664±0.610 | 1.088 | 0.283 |  |  |  |  |  |
|  | SVL max:Habitat Fossorial | 0.684±0.531 | 1.287 | 0.206 |  |  |  |  |  |
|  | SVL max:Habitat Terrestrial | 0.281±0.307 | 0.913 | 0.367 |  |  |  |  |  |
|  | Habitat Arboreal:Bio1 | -1.130±1.013 | -1.115 | 0.272 |  |  |  |  |  |
|  | Habitat Fossorial:Bio1 | 0.057±0.976 | 0.058 | 0.954 |  |  |  |  |  |
|  | Habitat Terrestrial:Bio1 | -0.104±0.410 | -0.253 | 0.802 |  |  |  |  |  |
|  | Habitat Arboreal:Bio4 | -0.254±0.342 | -0.743 | 0.462 |  |  |  |  |  |
|  | Habitat Fossorial:Bio4 | 0.025±1.716 | 0.015 | 0.988 |  |  |  |  |  |
|  | Habitat Terrestrial:Bio4 | 0.076±0.386 | 0.196 | 0.846 |  |  |  |  |  |
|  | Habitat Arboreal:Bio12 | 0.046±0.355 | 0.131 | 0.897 |  |  |  |  |  |
|  | Habitat Fossorial:Bio12 | 0.002±1.563 | 0.001 | 0.999 |  |  |  |  |  |
|  | Habitat Terrestrial:Bio12 | 0.131±0.272 | 0.481 | 0.633 |  |  |  |  |  |
|  | Habitat Arboreal:UVB1 | -0.326±1.187 | -0.275 | 0.785 |  |  |  |  |  |
|  | Habitat Fossorial:UVB1 | -0.468±2.749 | -0.170 | 0.866 |  |  |  |  |  |
|  | Habitat Terrestrial:UVB1 | 0.287±0.707 | 0.406 | 0.687 |  |  |  |  |  |
| Females | Intercept | 27.893±43.726 | 0.638 | 0.528 | 0.685 | (NA,0,241) | 2.174 | 0.000 | 0.015 |
|  | Bio1 | 1.879±11.558 | 0.163 | 0.872 |  |  |  |  |  |
|  | Bio4 | -3.939±6.592 | -0.598 | 0.554 |  |  |  |  |  |
|  | Bio12 | -6.456±8.026 | -0.804 | 0.427 |  |  |  |  |  |
|  | UVB1 | -8.635±11.312 | -0.763 | 0.451 |  |  |  |  |  |
|  | SVL max | 2.770±15.892 | 0.174 | 0.863 |  |  |  |  |  |
|  | Habitat Arboreal | 8.575±38.194 | 0.225 | 0.824 |  |  |  |  |  |
|  | Habitat Fossorial | -8.112±14.484 | -0.560 | 0.579 |  |  |  |  |  |
|  | Habitat Terrestrial | -2.341±4.792 | -0.489 | 0.628 |  |  |  |  |  |
|  | Bio1:Bio4 | 0.422±1.154 | 0.365 | 0.717 |  |  |  |  |  |
|  | Bio1:Bio12 | 0.108±1.285 | 0.084 | 0.933 |  |  |  |  |  |
|  | Bio1:UVB1 | -0.235±1.976 | -0.119 | 0.906 |  |  |  |  |  |
|  | SVL max:Bio1 | -1.385±0.923 | -1.500 | 0.143 |  |  |  |  |  |
|  | Bio4:Bio12 | -0.212±0.741 | -0.286 | 0.777 |  |  |  |  |  |
|  | Bio4:UVB1 | 1.525±1.701 | 0.896 | 0.377 |  |  |  |  |  |
|  | SVL max:Bio4 | -0.849±1.564 | -0.543 | 0.591 |  |  |  |  |  |
|  | Bio12:UVB1 | 1.624±2.171 | 0.748 | 0.460 |  |  |  |  |  |
|  | SVL max:Bio12 | 0.586±0.853 | 0.687 | 0.497 |  |  |  |  |  |
|  | SVL max:UVB1 | -0.107±3.023 | -0.036 | 0.972 |  |  |  |  |  |
|  | SVL max:Habitat Arboreal | 0.742±1.245 | 0.597 | 0.555 |  |  |  |  |  |
|  | SVL max:Habitat Fossorial | -0.117±0.790 | -0.149 | 0.883 |  |  |  |  |  |
|  | SVL max:Habitat Terrestrial | 0.341±0.299 | 1.138 | 0.263 |  |  |  |  |  |
|  | Habitat Arboreal:Bio1 | -1.013±2.126 | -0.477 | 0.637 |  |  |  |  |  |
|  | Habitat Fossorial:Bio1 | -1.223±1.018 | -1.201 | 0.238 |  |  |  |  |  |
|  | Habitat Terrestrial:Bio1 | -0.294±0.517 | -0.569 | 0.573 |  |  |  |  |  |
|  | Habitat Arboreal:Bio4 | -1.000±5.005 | -0.200 | 0.843 |  |  |  |  |  |
|  | Habitat Fossorial:Bio4 | 1.522±2.158 | 0.705 | 0.486 |  |  |  |  |  |
|  | Habitat Terrestrial:Bio4 | 0.161±0.590 | 0.272 | 0.787 |  |  |  |  |  |
|  | Habitat Arboreal:Bio12 | -0.244±2.224 | -0.110 | 0.913 |  |  |  |  |  |
|  | Habitat Fossorial:Bio12 | -1.102±1.657 | -0.665 | 0.511 |  |  |  |  |  |
|  | Habitat Terrestrial:Bio12 | 0.222±0.407 | 0.545 | 0.589 |  |  |  |  |  |
|  | Habitat Arboreal:UVB1 | -1.488±6.314 | -0.236 | 0.815 |  |  |  |  |  |
|  | Habitat Fossorial:UVB1 | 2.482±2.909 | 0.853 | 0.400 |  |  |  |  |  |
|  | Habitat Terrestrial:UVB1 | 0.273±0.819 | 0.333 | 0.741 |  |  |  |  |  |
| **Age at Sexual Maturity** | | | | | | | | | |
| Total | Intercept | 27.789±39.014 | 0.712 | 0.481 | 0.671 | (0.278,0.923) | 2.104 | 0.728 | 0.017 |
|  | Bio1 | 10.230±8.693 | 1.177 | 0.247 |  |  |  |  |  |
|  | Bio4 | -3.922±6.236 | -0.629 | 0.534 |  |  |  |  |  |
|  | Bio12 | -8.093±7.500 | -1.079 | 0.288 |  |  |  |  |  |
|  | UVB1 | -10.198±10.046 | -1.015 | 0.317 |  |  |  |  |  |
|  | SVL | -0.088±10.253 | -0.009 | 0.993 |  |  |  |  |  |
|  | Habitat Arboreal | 3.417±4.190 | 0.816 | 0.420 |  |  |  |  |  |
|  | Habitat Fossorial | 9.639±10.360 | 0.930 | 0.359 |  |  |  |  |  |
|  | Habitat Terrestrial | 2.133±3.033 | 0.703 | 0.487 |  |  |  |  |  |
|  | Bio1:Bio4 | -0.465±0.832 | -0.559 | 0.580 |  |  |  |  |  |
|  | Bio1:Bio12 | -1.910±1.040 | -1.837 | 0.075 |  |  |  |  |  |
|  | Bio1:UVB1 | -0.578±1.447 | -0.399 | 0.692 |  |  |  |  |  |
|  | Bio1:SVL | -0.755±0.733 | -1.030 | 0.310 |  |  |  |  |  |
|  | Bio1:Habitat Arboreal | 0.160±0.888 | 0.181 | 0.858 |  |  |  |  |  |
|  | Bio1:Habitat Fossorial | 1.299±0.805 | 1.615 | 0.116 |  |  |  |  |  |
|  | Bio1:Habitat Terrestrial | -0.156±0.410 | -0.379 | 0.707 |  |  |  |  |  |
|  | Bio4:Bio12 | -0.458±0.436 | -1.050 | 0.301 |  |  |  |  |  |
|  | Bio4:UVB1 | 1.528±1.537 | 0.994 | 0.327 |  |  |  |  |  |
|  | Bio4:SVL | 0.619±0.952 | 0.650 | 0.520 |  |  |  |  |  |
|  | Bio4:Habitat Arboreal | -0.356±0.278 | -1.283 | 0.208 |  |  |  |  |  |
|  | Bio4:Habitat Fossorial | -1.868±1.484 | -1.259 | 0.217 |  |  |  |  |  |
|  | Bio4:Habitat Terrestrial | -0.208±0.374 | -0.555 | 0.582 |  |  |  |  |  |
|  | Bio12:UVB1 | 2.930±2.039 | 1.437 | 0.160 |  |  |  |  |  |
|  | Bio12:SVL | 0.794±0.738 | 1.076 | 0.289 |  |  |  |  |  |
|  | Bio12:Habitat Arboreal | 0.148±0.315 | 0.470 | 0.641 |  |  |  |  |  |
|  | Bio12:Habitat Fossorial | 1.555±1.304 | 1.193 | 0.241 |  |  |  |  |  |
|  | Bio12:Habitat Terrestrial | 0.190±0.260 | 0.731 | 0.470 |  |  |  |  |  |
|  | UVB1:SVL | -0.843±2.052 | -0.411 | 0.684 |  |  |  |  |  |
|  | UVB1:Habitat Arboreal | -0.935±1.105 | -0.846 | 0.404 |  |  |  |  |  |
|  | UVB1:Habitat Fossorial | -4.093±2.219 | -1.844 | 0.074 |  |  |  |  |  |
|  | UVB1:Habitat Terrestrial | -0.615±0.582 | -1.057 | 0.298 |  |  |  |  |  |
|  | SVL:Habitat Arboreal | 0.132±0.517 | 0.256 | 0.799 |  |  |  |  |  |
|  | SVL:Habitat Fossorial | 2.036±0.563 | 3.618 | 0.001 |  |  |  |  |  |
|  | SVL:Habitat Terrestrial | 0.097±0.297 | 0.328 | 0.745 |  |  |  |  |  |
| Males | Intercept | 17.977±56.479 | 0.318 | 0.753 | 0.588 | (0.282,0.874) | 1.252 | 0.680 | 0.271 |
|  | Bio1 | 7.134±10.874 | 0.656 | 0.517 |  |  |  |  |  |
|  | Bio4 | -3.433±8.317 | -0.413 | 0.683 |  |  |  |  |  |
|  | Bio12 | -5.649±8.037 | -0.703 | 0.488 |  |  |  |  |  |
|  | UVB1 | -6.792±13.643 | -0.498 | 0.622 |  |  |  |  |  |
|  | SVL | 1.855±18.412 | 0.101 | 0.920 |  |  |  |  |  |
|  | Habitat Arboreal | 2.417±5.008 | 0.483 | 0.633 |  |  |  |  |  |
|  | Habitat Fossorial | 14.234±12.362 | 1.151 | 0.259 |  |  |  |  |  |
|  | Habitat Terrestrial | 1.798±4.735 | 0.380 | 0.707 |  |  |  |  |  |
|  | Bio1:Bio4 | -0.106±1.251 | -0.085 | 0.933 |  |  |  |  |  |
|  | Bio1:Bio12 | -1.500±1.168 | -1.284 | 0.209 |  |  |  |  |  |
|  | Bio1:UVB1 | -0.378±1.786 | -0.211 | 0.834 |  |  |  |  |  |
|  | Bio1:SVL | -0.685±1.013 | -0.676 | 0.505 |  |  |  |  |  |
|  | Bio1:Habitat Arboreal | 0.316±0.977 | 0.323 | 0.749 |  |  |  |  |  |
|  | Bio1:Habitat Fossorial | 1.356±0.973 | 1.394 | 0.174 |  |  |  |  |  |
|  | Bio1:Habitat Terrestrial | -0.297±0.415 | -0.716 | 0.479 |  |  |  |  |  |
|  | Bio4:Bio12 | -0.590±0.684 | -0.862 | 0.396 |  |  |  |  |  |
|  | Bio4:UVB1 | 1.375±1.818 | 0.756 | 0.456 |  |  |  |  |  |
|  | Bio4:SVL | 0.544±1.973 | 0.276 | 0.785 |  |  |  |  |  |
|  | Bio4:Habitat Arboreal | -0.206±0.425 | -0.483 | 0.633 |  |  |  |  |  |
|  | Bio4:Habitat Fossorial | -2.267±1.801 | -1.259 | 0.218 |  |  |  |  |  |
|  | Bio4:Habitat Terrestrial | -0.224±0.593 | -0.378 | 0.708 |  |  |  |  |  |
|  | Bio12:UVB1 | 2.159±2.203 | 0.980 | 0.335 |  |  |  |  |  |
|  | Bio12:SVL | 0.865±1.033 | 0.838 | 0.409 |  |  |  |  |  |
|  | Bio12:Habitat Arboreal | 0.107±0.382 | 0.281 | 0.781 |  |  |  |  |  |
|  | Bio12:Habitat Fossorial | 1.311±1.601 | 0.819 | 0.420 |  |  |  |  |  |
|  | Bio12:Habitat Terrestrial | 0.260±0.358 | 0.727 | 0.473 |  |  |  |  |  |
|  | Uvb1:SVL | -1.418±3.075 | -0.461 | 0.648 |  |  |  |  |  |
|  | Uvb1:Habitat Arboreal | -0.696±1.188 | -0.586 | 0.562 |  |  |  |  |  |
|  | Uvb1:Habitat Fossorial | -4.658±2.520 | -1.849 | 0.075 |  |  |  |  |  |
|  | Uvb1:Habitat Terrestrial | -0.538±0.757 | -0.711 | 0.483 |  |  |  |  |  |
|  | SVL:Habitat Arboreal | -0.050±0.591 | -0.085 | 0.933 |  |  |  |  |  |
|  | SVL:Habitat Fossorial | 1.609±0.613 | 2.623 | 0.014 |  |  |  |  |  |
|  | SVL:Habitat Terrestrial | 0.131±0.350 | 0.375 | 0.711 |  |  |  |  |  |
| Females | Intercept | 10.407±43.559 | 0.239 | 0.813 | 0.626 | (0.134,0.888) | 1.472 | 0.650 | 0.015 |
|  | Bio1 | 11.188±9.956 | 1.124 | 0.270 |  |  |  |  |  |
|  | Bio4 | 0.946±6.791 | 0.139 | 0.890 |  |  |  |  |  |
|  | Bio12 | -5.692±8.302 | -0.686 | 0.498 |  |  |  |  |  |
|  | UVB1 | -6.061±11.115 | -0.545 | 0.590 |  |  |  |  |  |
|  | SVL | -0.475±12.472 | -0.038 | 0.970 |  |  |  |  |  |
|  | Habitat Arboreal | 1.489±4.379 | 0.340 | 0.736 |  |  |  |  |  |
|  | Habitat Fossorial | 8.048±11.514 | 0.699 | 0.490 |  |  |  |  |  |
|  | Habitat Terrestrial | 1.532±3.184 | 0.481 | 0.634 |  |  |  |  |  |
|  | Bio1:Bio4 | -0.227±1.004 | -0.226 | 0.823 |  |  |  |  |  |
|  | Bio1:Bio12 | -1.651±1.103 | -1.496 | 0.145 |  |  |  |  |  |
|  | Bio1:UVB1 | -0.909±1.661 | -0.547 | 0.588 |  |  |  |  |  |
|  | Bio1:SVL | -1.368±1.682 | -0.813 | 0.423 |  |  |  |  |  |
|  | Bio1:Habitat Arboreal | 0.040±0.944 | 0.042 | 0.967 |  |  |  |  |  |
|  | Bio1:Habitat Fossorial | 0.690±0.865 | 0.798 | 0.431 |  |  |  |  |  |
|  | Bio1:Habitat Terrestrial | -0.144±0.476 | -0.301 | 0.765 |  |  |  |  |  |
|  | Bio4:Bio12 | -0.646±0.487 | -1.328 | 0.194 |  |  |  |  |  |
|  | Bio4:UVB1 | 0.371±1.670 | 0.222 | 0.826 |  |  |  |  |  |
|  | Bio4:SVL | 0.287±1.101 | 0.261 | 0.796 |  |  |  |  |  |
|  | Bio4:Habitat Arboreal | -0.175±0.389 | -0.451 | 0.655 |  |  |  |  |  |
|  | Bio4:Habitat Fossorial | -1.890±1.712 | -1.104 | 0.279 |  |  |  |  |  |
|  | Bio4:Habitat Terrestrial | -0.064±0.393 | -0.163 | 0.872 |  |  |  |  |  |
|  | Bio12:UVB1 | 2.313±2.230 | 1.037 | 0.308 |  |  |  |  |  |
|  | Bio12:SVL | 0.763±0.816 | 0.935 | 0.358 |  |  |  |  |  |
|  | Bio12:Habitat Arboreal | 0.336±0.486 | 0.691 | 0.495 |  |  |  |  |  |
|  | Bio12:Habitat Fossorial | 0.958±1.322 | 0.725 | 0.474 |  |  |  |  |  |
|  | Bio12:Habitat Terrestrial | 0.296±0.294 | 1.006 | 0.323 |  |  |  |  |  |
|  | UVB1:SVL | -0.233±2.757 | -0.085 | 0.933 |  |  |  |  |  |
|  | UVB1:Habitat Arboreal | -0.712±1.204 | -0.592 | 0.559 |  |  |  |  |  |
|  | UVB1:Habitat Fossorial | -2.773±2.338 | -1.186 | 0.245 |  |  |  |  |  |
|  | UVB1:Habitat Terrestrial | -0.588±0.606 | -0.970 | 0.340 |  |  |  |  |  |
|  | SVL:Habitat Arboreal | 0.254±0.744 | 0.341 | 0.736 |  |  |  |  |  |
|  | SVL:Habitat Fossorial | 1.723±0.723 | 2.381 | 0.024 |  |  |  |  |  |
|  | SVL:Habitat Terrestrial | -0.016±0.293 | -0.053 | 0.958 |  |  |  |  |  |
| **Average age** | | | | | | | | | |
| Total | Intercept | 31.337±27.883 | 1.124 | 0.266 | 0.013 | (NA,0.908) | 21.967 | 0.663 | 0.013 |
|  | Bio1 | 13.857±7.976 | 1.737 | 0.088 |  |  |  |  |  |
|  | Bio4 | -3.880±4.607 | -0.842 | 0.403 |  |  |  |  |  |
|  | Bio12 | -11.309±5.469 | -2.068 | 0.043 |  |  |  |  |  |
|  | UVB1 | -10.799±6.889 | -1.568 | 0.123 |  |  |  |  |  |
|  | SVL | 1.299±7.592 | 0.171 | 0.865 |  |  |  |  |  |
|  | Habitat Arboreal | 4.248±2.519 | 1.686 | 0.097 |  |  |  |  |  |
|  | Habitat Fossorial | -1.785±8.793 | -0.203 | 0.840 |  |  |  |  |  |
|  | Habitat Terrestrial | -0.186±2.637 | -0.070 | 0.944 |  |  |  |  |  |
|  | Bio1:Bio4 | -0.452±0.837 | -0.540 | 0.591 |  |  |  |  |  |
|  | Bio1:Bio12 | -1.503±0.758 | -1.983 | 0.052 |  |  |  |  |  |
|  | Bio1:UVB1 | -1.761±1.325 | -1.329 | 0.189 |  |  |  |  |  |
|  | Bio1:SVL | -1.036±0.853 | -1.214 | 0.230 |  |  |  |  |  |
|  | Bio1:Habitat Arboreal | -0.767±0.538 | -1.426 | 0.159 |  |  |  |  |  |
|  | Bio1:Habitat Fossorial | 0.101±0.600 | 0.168 | 0.867 |  |  |  |  |  |
|  | Bio1:Habitat Terrestrial | -0.539±0.362 | -1.492 | 0.142 |  |  |  |  |  |
|  | Bio4:Bio12 | -0.170±0.391 | -0.435 | 0.665 |  |  |  |  |  |
|  | Bio4:UVB1 | 1.432±1.132 | 1.265 | 0.211 |  |  |  |  |  |
|  | Bio4:SVL | 0.137±0.769 | 0.179 | 0.859 |  |  |  |  |  |
|  | Bio4:Habitat Arboreal | -0.421±0.234 | -1.804 | 0.077 |  |  |  |  |  |
|  | Bio4:Habitat Fossorial | -0.089±1.247 | -0.071 | 0.944 |  |  |  |  |  |
|  | Bio4:Habitat Terrestrial | -0.002±0.317 | -0.008 | 0.994 |  |  |  |  |  |
|  | Bio12:UVB1 | 3.511±1.429 | 2.456 | 0.017 |  |  |  |  |  |
|  | Bio12:SVL | 0.822±0.588 | 1.398 | 0.168 |  |  |  |  |  |
|  | Bio12:Habitat Arboreal | -0.065±0.267 | -0.245 | 0.807 |  |  |  |  |  |
|  | Bio12:Habitat Fossorial | 0.789±0.746 | 1.057 | 0.295 |  |  |  |  |  |
|  | Bio12:Habitat Terrestrial | -0.158±0.191 | -0.825 | 0.413 |  |  |  |  |  |
|  | UVB1:SVL | -0.809±1.468 | -0.551 | 0.584 |  |  |  |  |  |
|  | UVB1:Habitat Arboreal | -0.899±0.514 | -1.749 | 0.086 |  |  |  |  |  |
|  | UVB1:Habitat Fossorial | -0.637±2.017 | -0.316 | 0.754 |  |  |  |  |  |
|  | UVB1:Habitat Terrestrial | 0.175±0.504 | 0.348 | 0.729 |  |  |  |  |  |
|  | SVL:Habitat Arboreal | 0.686±0.444 | 1.546 | 0.128 |  |  |  |  |  |
|  | SVL:Habitat Fossorial | 1.111±0.481 | 2.308 | 0.025 |  |  |  |  |  |
|  | SVL:Habitat Terrestrial | 0.369±0.280 | 1.317 | 0.193 |  |  |  |  |  |
| Males | Intercept | 62.193±31.402 | 1.981 | 0.053 | 0.725 | (NA,0.675) | 4.068 | 0.000 | 0.271 |
|  | Bio1 | 15.791±9.253 | 1.707 | 0.094 |  |  |  |  |  |
|  | Bio4 | -6.728±5.292 | -1.271 | 0.209 |  |  |  |  |  |
|  | Bio12 | -19.192±6.260 | -3.066 | 0.003 |  |  |  |  |  |
|  | UVB1 | -19.982±7.920 | -2.523 | 0.015 |  |  |  |  |  |
|  | SVL | 1.001±8.535 | 0.117 | 0.907 |  |  |  |  |  |
|  | Habitat Arboreal | 5.067±2.954 | 1.716 | 0.092 |  |  |  |  |  |
|  | Habitat Fossorial | 3.346±9.925 | 0.337 | 0.737 |  |  |  |  |  |
|  | Habitat Terrestrial | -1.851±3.338 | -0.555 | 0.582 |  |  |  |  |  |
|  | Bio1:Bio4 | -1.082±0.983 | -1.101 | 0.276 |  |  |  |  |  |
|  | Bio1:Bio12 | -2.290±0.902 | -2.538 | 0.014 |  |  |  |  |  |
|  | Bio1:UVB1 | -1.398±1.522 | -0.919 | 0.362 |  |  |  |  |  |
|  | Bio1:SVL | -0.494±0.901 | -0.548 | 0.586 |  |  |  |  |  |
|  | Bio1:Habitat Arboreal | -0.752±0.602 | -1.250 | 0.217 |  |  |  |  |  |
|  | Bio1:Habitat Fossorial | 0.445±0.672 | 0.663 | 0.511 |  |  |  |  |  |
|  | Bio1:Habitat Terrestrial | -0.397±0.450 | -0.882 | 0.382 |  |  |  |  |  |
|  | Bio4:Bio12 | -0.014±0.453 | -0.032 | 0.975 |  |  |  |  |  |
|  | Bio4:Uvb1 | 2.269±1.303 | 1.741 | 0.088 |  |  |  |  |  |
|  | Bio4:SVL | 0.190±0.856 | 0.222 | 0.825 |  |  |  |  |  |
|  | Bio4:Habitat Arboreal | -0.384±0.281 | -1.364 | 0.179 |  |  |  |  |  |
|  | Bio4:Habitat Fossorial | -0.442±1.427 | -0.310 | 0.758 |  |  |  |  |  |
|  | Bio4:Habitat Terrestrial | 0.304±0.391 | 0.777 | 0.440 |  |  |  |  |  |
|  | Bio12:Uvb1 | 5.819±1.675 | 3.474 | 0.001 |  |  |  |  |  |
|  | Bio12:SVL | 0.903±0.670 | 1.349 | 0.183 |  |  |  |  |  |
|  | Bio12:Habitat Arboreal | -0.029±0.308 | -0.095 | 0.925 |  |  |  |  |  |
|  | Bio12:Habitat Fossorial | 1.083±0.747 | 1.449 | 0.154 |  |  |  |  |  |
|  | Bio12:Habitat Terrestrial | -0.030±0.228 | -0.132 | 0.896 |  |  |  |  |  |
|  | Uvb1:SVL | -0.993±1.656 | -0.600 | 0.551 |  |  |  |  |  |
|  | Uvb1:Habitat Arboreal | -1.113±0.624 | -1.785 | 0.080 |  |  |  |  |  |
|  | Uvb1:Habitat Fossorial | -2.222±2.357 | -0.943 | 0.350 |  |  |  |  |  |
|  | Uvb1:Habitat Terrestrial | 0.314±0.645 | 0.486 | 0.629 |  |  |  |  |  |
|  | SVL:Habitat Arboreal | 0.534±0.510 | 1.047 | 0.300 |  |  |  |  |  |
|  | SVL:Habitat Fossorial | 1.249±0.462 | 2.705 | 0.009 |  |  |  |  |  |
|  | SVL:Habitat Terrestrial | 0.264±0.280 | 0.942 | 0.350 |  |  |  |  |  |
| Females | Intercept | 26.881±30.105 | 0.893 | 0.377 | 0.488 | (0.287,0.993) | 1.302 | 0.899 | 0.203 |
|  | Bio1 | 5.789±9.096 | 0.636 | 0.528 |  |  |  |  |  |
|  | Bio4 | -5.176±4.900 | -1.056 | 0.296 |  |  |  |  |  |
|  | Bio12 | -6.601±5.798 | -1.138 | 0.261 |  |  |  |  |  |
|  | Uvb1 | -6.748±7.298 | -0.925 | 0.360 |  |  |  |  |  |
|  | SVL | -2.160±9.171 | -0.236 | 0.815 |  |  |  |  |  |
|  | Habitat Arboreal | 4.255±3.005 | 1.416 | 0.164 |  |  |  |  |  |
|  | Habitat Fossorial | -6.206±9.462 | -0.656 | 0.515 |  |  |  |  |  |
|  | Habitat Terrestrial | 3.100±2.605 | 1.190 | 0.240 |  |  |  |  |  |
|  | Bio1:Bio4 | 0.467±0.938 | 0.498 | 0.621 |  |  |  |  |  |
|  | Bio1:Bio12 | -0.401±0.803 | -0.499 | 0.620 |  |  |  |  |  |
|  | Bio1:Uvb1 | -1.007±1.498 | -0.672 | 0.505 |  |  |  |  |  |
|  | Bio1:SVL | -1.309±0.935 | -1.400 | 0.168 |  |  |  |  |  |
|  | Bio1:Habitat Arboreal | -0.494±0.733 | -0.674 | 0.504 |  |  |  |  |  |
|  | Bio1:Habitat Fossorial | -0.411±0.653 | -0.629 | 0.532 |  |  |  |  |  |
|  | Bio1:Habitat Terrestrial | -0.665±0.358 | -1.861 | 0.069 |  |  |  |  |  |
|  | Bio4:Bio12 | -0.207±0.419 | -0.493 | 0.624 |  |  |  |  |  |
|  | Bio4:Uvb1 | 1.406±1.196 | 1.176 | 0.246 |  |  |  |  |  |
|  | Bio4:SVL | 0.339±0.842 | 0.402 | 0.689 |  |  |  |  |  |
|  | Bio4:Habitat Arboreal | -0.271±0.285 | -0.950 | 0.347 |  |  |  |  |  |
|  | Bio4:Habitat Fossorial | 0.625±1.322 | 0.473 | 0.639 |  |  |  |  |  |
|  | Bio4:Habitat Terrestrial | -0.472±0.319 | -1.479 | 0.146 |  |  |  |  |  |
|  | Bio12:Uvb1 | 1.587±1.548 | 1.026 | 0.311 |  |  |  |  |  |
|  | Bio12:SVL | 1.364±0.637 | 2.141 | 0.038 |  |  |  |  |  |
|  | Bio12:HabitatArboreal | -0.024±0.497 | -0.049 | 0.961 |  |  |  |  |  |
|  | Bio12:Habitat Fossorial | -0.130±0.853 | -0.152 | 0.880 |  |  |  |  |  |
|  | Bio12:Habitat Terrestrial | -0.248±0.202 | -1.227 | 0.226 |  |  |  |  |  |
|  | Uvb1:SVL | -0.352±1.815 | -0.194 | 0.847 |  |  |  |  |  |
|  | Uvb1:Habitat Arboreal | -0.934±0.558 | -1.674 | 0.101 |  |  |  |  |  |
|  | Uvb1:Habitat Fossorial | 1.203±2.113 | 0.569 | 0.572 |  |  |  |  |  |
|  | Uvb1:Habitat Terrestrial | -0.219±0.514 | -0.426 | 0.672 |  |  |  |  |  |
|  | SVL:Habitat Arboreal | 0.281±0.632 | 0.445 | 0.658 |  |  |  |  |  |
|  | SVL:Habitat Fossorial | 0.677±0.634 | 1.069 | 0.291 |  |  |  |  |  |
|  | SVL:Habitat Terrestrial | 0.286±0.312 | 0.917 | 0.364 |  |  |  |  |  |

Notes: CI, confidence interval; SVL, snout–vent length. The *p*-value indicates the level of significance of phylogenetic signals

**Table S3** Association outcomes between age-structure traits and geographical drivers based on phylogenetic pathway analysis.

|  | **model** | ***k*** | ***q*** | ***C*** | ***CICc*** | ***delta_CICc*** | ***wi*** |
| --- | --- | --- | --- | --- | --- | --- | --- |
| **m12** | **m12** | **9** | **12** | **18.682** | **48.682** | **0.000** | **0.364** |
| **m6** | **m6** | **9** | **12** | **18.807** | **48.807** | **0.125** | **0.342** |
| **m8** | **m8** | **9** | **12** | **19.743** | **49.743** | **1.061** | **0.214** |
| m9 | m9 | 8 | 13 | 18.966 | 52.104 | 3.421 | 0.066 |
| m11 | m11 | 10 | 11 | 29.068 | 56.049 | 7.367 | 0.009 |
| m10 | m10 | 9 | 12 | 28.721 | 58.721 | 10.038 | 0.002 |
| m5 | m5 | 8 | 13 | 27.349 | 60.486 | 11.804 | 0.001 |
| m14 | m14 | 7 | 14 | 25.928 | 62.328 | 13.646 | 0.000 |
| m1 | m1 | 7 | 14 | 26.440 | 62.840 | 14.158 | 0.000 |
| m3 | m3 | 8 | 13 | 30.451 | 63.588 | 14.906 | 0.000 |
| m15 | m15 | 10 | 11 | 38.231 | 65.212 | 16.530 | 0.000 |
| m7 | m7 | 9 | 12 | 36.563 | 66.563 | 17.881 | 0.000 |
| m4 | m4 | 8 | 13 | 36.697 | 69.834 | 21.152 | 0.000 |
| m2 | m2 | 8 | 13 | 37.315 | 48.682 | 21.770 | 0.000 |
| m13 | m13 | 8 | 13 | 39.157 | 48.807 | 23.612 | 0.000 |

Notes: *k* = number of independence claims; *q* = number of parameters; *C* = Fisher’s C statistics; *CICc* = C-statistic Information Criterion; *delta_CICc* = difference in CICc from the best-fitting model; *wi* = CICc weights.

**Table S4** Association outcomes between age-structure traits and climatic drivers based on phylogenetic pathway analysis.

|  | model | *k* | ***q*** | *C* | ***CICc*** | *delta_CICc* | *wi* |
| --- | --- | --- | --- | --- | --- | --- | --- |
| **m4** | **m4** | **19** | **17** | **93.094** | **140.116** | **0.000** | **0.418** |
| **m7** | **m7** | **20** | **16** | **97.470** | **140.803** | **0.687** | **0.296** |
| **m2** | **m2** | **18** | **18** | **90.080** | **140.949** | **0.833** | **0.275** |
| m14 | m14 | 19 | 17 | 100.389 | 147.410 | 7.294 | 0.01 |
| m15 | m15 | 20 | 16 | 118.398 | 161.731 | 21.615 | 0.000 |
| m3 | m3 | 19 | 17 | 117.334 | 164.356 | 24.240 | 0.000 |
| m5 | m5 | 19 | 17 | 117.992 | 165.013 | 24.897 | 0.000 |
| m12 | m12 | 22 | 14 | 130.505 | 166.905 | 26.789 | 0.000 |
| m8 | m8 | 21 | 15 | 127.992 | 167.788 | 27.672 | 0.000 |
| m10 | m10 | 20 | 16 | 125.387 | 168.720 | 28.605 | 0.000 |
| m9 | m9 | 19 | 17 | 123.524 | 170.546 | 30.430 | 0.000 |
| m13 | m13 | 20 | 16 | 127.749 | 171.082 | 30.966 | 0.000 |
| m6 | m6 | 20 | 16 | 127.785 | 171.118 | 31.002 | 0.000 |

Notes: *k* = number of independence claims; *q* = number of parameters; *C* = Fisher’s C statistics; *CICc* = C-statistic Information Criterion; *delta_CICc* = difference in CICc from the best-fitting model; *wi* = CICc weights.
